# Adaptive selection of a prion strain conformer corresponding to established North American CWD during propagation of novel emergent Norwegian strains in mice expressing elk or deer prion protein

**DOI:** 10.1371/journal.ppat.1009748

**Published:** 2021-07-26

**Authors:** Jifeng Bian, Sehun Kim, Sarah J. Kane, Jenna Crowell, Julianna L. Sun, Jeffrey Christiansen, Eri Saijo, Julie A. Moreno, James DiLisio, Emily Burnett, Sandra Pritzkow, Damian Gorski, Claudio Soto, Terry J. Kreeger, Aru Balachandran, Gordon Mitchell, Michael W. Miller, Romolo Nonno, Turid Vikøren, Jørn Våge, Knut Madslien, Linh Tran, Tram Thu Vuong, Sylvie L. Benestad, Glenn C. Telling

**Affiliations:** 1 Prion Research Center (PRC), the Department of Microbiology, Immunology and Pathology, Colorado State University, Fort Collins, Colorado, United States of America; 2 Program in Cell and Molecular Biology, Colorado State University, Fort Collins, Colorado, United States of America; 3 Mitchell Center for Alzheimer’s Disease and Related Brain Disorders, Department of Neurology, University of Texas Houston Medical School, Houston, Texas, United States of America; 4 Wyoming Game and Fish Department, Wheatland, Wyoming, United States of America; 5 Canadian Food Inspection Agency, National and OIE Reference Laboratory for Scrapie and CWD, Ottawa, Canada; 6 Colorado Parks and Wildlife, Fort Collins, Colorado, United States of America; 7 Istituto Superiore di Sanità, Department of Veterinary Public Health, Nutrition and Food Safety, Rome, Italy; 8 Norwegian Veterinary Institute, OIE Reference laboratory for CWD, Oslo, Norway; Dartmouth College Geisel School of Medicine, UNITED STATES

## Abstract

Prions are infectious proteins causing fatal, transmissible neurodegenerative diseases of animals and humans. Replication involves template-directed refolding of host encoded prion protein, PrP^C^, by its infectious conformation, PrP^Sc^. Following its discovery in captive Colorado deer in 1967, uncontrollable contagious transmission of chronic wasting disease (CWD) led to an expanded geographic range in increasing numbers of free-ranging and captive North American (NA) cervids. Some five decades later, detection of PrP^Sc^ in free-ranging Norwegian (NO) reindeer and moose marked the first indication of CWD in Europe. To assess the properties of these emergent NO prions and compare them with NA CWD we used transgenic (Tg) and gene targeted (Gt) mice expressing PrP with glutamine (Q) or glutamate (E) at residue 226, a variation in wild type cervid PrP which influences prion strain selection in NA deer and elk. Transmissions of NO moose and reindeer prions to Tg and Gt mice recapitulated the characteristic features of CWD in natural hosts, revealing novel prion strains with disease kinetics, neuropathological profiles, and capacities to infect lymphoid tissues and cultured cells that were distinct from those causing NA CWD. In support of strain variation, PrP^Sc^ conformers comprising emergent NO moose and reindeer CWD were subject to selective effects imposed by variation at residue 226 that were different from those controlling established NA CWD. Transmission of particular NO moose CWD prions in mice expressing E at 226 resulted in selection of a kinetically optimized conformer, subsequent transmission of which revealed properties consistent with NA CWD. These findings illustrate the potential for adaptive selection of strain conformers with improved fitness during propagation of unstable NO prions. Their potential for contagious transmission has implications for risk analyses and management of emergent European CWD. Finally, we found that Gt mice expressing physiologically controlled PrP levels recapitulated the lymphotropic properties of naturally occurring CWD strains resulting in improved susceptibilities to emergent NO reindeer prions compared with over-expressing Tg counterparts. These findings underscore the refined advantages of Gt models for exploring the mechanisms and impacts of strain selection in peripheral compartments during natural prion transmission.

## Author summary

Prions cause fatal, transmissible neurodegenerative diseases in animals and humans. They are composed of an infectious, neurotoxic protein (PrP) which replicates by imposing pathogenic conformations on its normal, host-encoded counterpart. Chronic wasting disease (CWD) is a contagious prion disorder threatening increasing numbers of free-ranging and captive North American deer, elk, and moose. While CWD detection in Norwegian reindeer and moose in 2016 marked the advent of disease in Europe, its origins and relationship to North American CWD were initially unclear. Here we show, using mice engineered to express deer or elk PrP, that Norwegian reindeer and moose CWD are caused by novel prion strains with properties distinct from those of North American CWD. We found that selection and propagation of North American and Norwegian CWD strains was controlled by a key amino acid residue in host PrP. We also found that particular Norwegian isolates adapted during their propagation in mice to produce prions with characteristics of the North American strain. Our findings defining the transmission profiles of novel Norwegian prions and their unstable potential to produce adapted strains with improved fitness for contagious transmission have implications for risk analyses and management of emergent European CWD.

## Introduction

Prions are infectious proteins that cause inexorable degenerative diseases of the central nervous system (CNS) in humans and animals. The replicative properties of prion proteins and the mechanisms by which they mediate information transfer in biological systems challenge fundamental concepts of inheritance and pathogenesis. Prions are composed of PrP^Sc^, a relatively under glycosylated [1] and conformationally altered [2] counterpart of normal host-encoded cellular PrP (PrP^C^). During disease PrP^Sc^ interacts with and imparts its infective β-sheet conformation on α-helical PrP^C^ by template-directed refolding. The upshot of this cyclical process is exponential prion accumulation, resultant neurodegeneration, and inevitable death [3].

Although they lack informational nucleic acids, prions share with conventional pathogens the property of heritable and mutable strain diversity [4]. Strain properties influence disease outcomes including the period of latency between infection and eventual onset of clinical signs and symptoms, as well as the targeting of prions to defined neuronal populations in the CNS [5] and non-CNS tissues, particularly those of the lymphoreticular system (LRS) and musculature [6–9]. Strain characteristics are also important when considering the potential for and outcomes of interspecies prion transmission. Strain properties define prion host range potentials [10] and although full or even partial acclimatization is not an obligatory consequence of disease transmission to a new host species [11], strain adaptation is nonetheless a customary outcome [12,13]. The efficiency of this process is dictated by several parameters which influence the interplay between PrP^Sc^ constituting infectious prions and host encoded PrP^C^. First, heritable strain information is enciphered by distinct PrP^Sc^ conformations [14,15]; second, strain adaptation following interspecies transmission to a new host [16] or in response to other selective pressures [17] is associated with concomitant changes in PrP^Sc^ conformation; and third, prion transmission between species is facilitated when primary structures of PrP^Sc^ constituting the infectious agent match those of PrP^C^ expressed in the newly infected host species [18].

While scrapie of sheep and goats and Creutzfeldt Jakob disease (CJD) of humans [19] are considered archetypal prion diseases, recent decades have witnessed occurrences of novel disorders and strain variants of uncertain origin and epidemic potential in increasing numbers of species, the most recently discovered example being a prion disease of North African dromedaries [20]. The emergence of bovine spongiform encephalopathy (BSE) and its transmission to humans to produce a variant of CJD (vCJD) [10] epitomize the unpredictable epidemiology of prion diseases, their potentially devastating economic and societal effects, and the catastrophic public health consequences of prion zoonoses. Today chronic wasting disease (CWD), an uncontrollably contagious and ineradicable prion disease of free-ranging and captive cervids, presents a growing ecological threat and a possible danger to human health. Initially identified in Colorado in 1967 and subsequently thought to be restricted to a relatively small endemic region comprising Northern Colorado and South Eastern Wyoming, the recognized geographic and host ranges of CWD have expanded to such an extent that it now jeopardizes the survival of increasing numbers of cervid populations and species across North America (NA). Twenty-six states and three Canadian provinces have reported disease in farmed and/or free ranging species of mule deer (*Odocoileus hemionus*), white tailed deer (*O*. *virginianus*), elk (*Cervus canadensis*), and Shiras’ moose (*Alces alces shirasi*). Many but not all of the temporally-and spatially-dispersed occurrences documented in NA since the 1960s can be connected via inadvertent movements of infected animals in commerce. In 2016 PrP^Sc^ was detected in the CNS of a free-ranging Norwegian (NO) reindeer (*Rangifer tarandus*), marking the first indication of CWD in Europe or in this species [21]. Further testing identified PrP^Sc^ in additional NO reindeer [22], in growing numbers of European moose (*Alces alces alces*) (also known as European elk) [23], and in red deer (*Cervus elaphus*) [24]. CWD cases were also subsequently diagnosed in moose from Sweden and Finland [25]. Additional at-risk free-ranging cervid species in Europe include Roe deer (*C*. *capreolus*), fallow deer (*Dama dama*), sika deer (*C*. *nippon*), Chinese water deer (*Hydropotes inermis*), and Reeves’s muntjac (*Muntiacus reevesi*) (Table 1) [26].

**Table 1:** PrP codon 226 genotypes of CWD-affected and at-risk European cervid species and recipients used for experimental transmissions.

**Recipients used in transmission experiments**		
Recipients	PrP codon 226	PrP expression level
TgQ226	Q^1^	~ 5-fold > wild type mice
TgE226	E^1^	~ 5-fold > wild type mice
GtQ226	Q/Q^2^	Equal to wild type mice
GtE226	E/E^2^	Equal to wild type mice
Bank vole	Q/Q^3^	Wild type
**CWD transmissions to Tg and Gt mice in previous studies**		
Species	PrP codon 226	Origin
Elk (*C*. *canadensis*)	E/E	North America
Mule deer (*O*. *hemionus*)	Q/Q	North America
White tailed deer (*O*. *virginianus*)	Q/Q	North America
**CWD transmissions to Tg and Gt mice in the present study**		
Species	PrP codon 226	Origin
Moose (*A*. *a*. *shirasi*)	Q/Q	North America
Moose (*A*. *a*. *alces*)	Q/Q	Norway
Reindeer (*R*. *tarandus*)	Q/Q	Norway
**Additional European cervid species**^4^		
Species	PrP codon 226	
Red deer (*C*. *elaphus*)^5^	E/E, Q/Q, E/Q	
Roe deer (*C*. *capreolus*)	Q/Q	
Fallow deer (*D*. *dama*)	E/E	
Reeves’s muntjac (*M*. *reevesi*)	Q/Q	
Sika deer (*C*. *nippon*)	Q/Q	

^1^ Transgenes in TgQ226 and TgE226 mice are composed of tandem arrays of multiple expression cassettes resulting in overexpression of Q226 or E226 respectively. Since transgenes are randomly-integrated at single undefined loci in the mouse genome, mice are described as hemizygous for these respective transgene arrays.

^2^ In GtQ226 and GtE226 mice the murine PrP coding sequence was replaced with Q226 or E226 and are therefore homozygous (Q/Q or E/E) for the targeted *Prnp* locus.

^3^ Codon 223 in the bank vole PrP coding sequence

^4^ See [26]

^5^ CWD diagnosed in Norwegian red deer [24]

Seminal studies in transgenic (Tg) mice underscored the importance of PrP sequence identity for facilitating interspecies prion transmission [18,27–29]. This finding motivated the development of tractable experimental models which revolutionized studies of human and animal prion diseases by eliminating barriers to their transmission as well as associated requirements for strain adaptation in a new species [30]. The PrP coding sequences of CWD-susceptible cervid species (CerPrP) vary at codon 226: whereas deer, reindeer and moose express CerPrP^C^ with glutamine at residue 226, elk express glutamate (abbreviated here as Q226 and E226 respectively) (Table 1) or, because of heterozygosity, are dimorphic at this position; red deer may be homozygous for Q226 or E226, or heterozygous [26,31–34]. We previously reported that prototype CWD-susceptible Tg mice which over-express Q226 or E226 in the CNS (Table 1), hereafter referred to as TgQ226 and TgE226, were susceptible following intracerebral (ic) challenges with CWD prions from the CNS, LRS, skeletal muscles and other tissues of NA deer and elk with CWD [9,29,35–37]. The unaltered properties of CWD prions propagated in TgQ226 mice following their retransmission to white-tailed deer provided definitive verification that Tg mice provided an authentic system in which to define the prevalence and properties of NA CWD strains [36,38]. Gene-targeted (Gt) mice in which the murine PrP coding sequence was replaced with Q226 or E226, referred to as GtQ226 and GtE226 (Table 1), provided the refined advantages of physiologically matched expression of Q226 or E226 in an otherwise invariant genetic background and the ability to recapitulate additional cardinal features of CWD in natural hosts including authentic tissue peripheralization and contagious propagation [39]. Transmission of NA deer and elk CWD isolates to Tg and Gt mice showed that natural variation of Q and E at residue 226 controls the selection and propagation of distinct NA CWD prion strains [39].

While it was initially unclear whether emergent cases of Scandinavian CWD originated from NA CWD as occurred following commercial trade and export of subclinically diseased NA elk to South Korea [40–42], our collaborative studies conducted in parallel to those reported here using bank voles (BV), a species previously shown to be highly susceptible to NA CWD [43], provided the first indication that the properties of NO CWD prions are unrelated to NA CWD [44]. This observation lent credence to the possibility that all CWD foci are not necessarily interconnected and that the circumstances minimally giving rise to outbreaks in Colorado and NO may have been (or could be) replicated over time in other locations. Here we used CWD-susceptible Tg and Gt mice expressing Q226 or E226 to comprehensively assess the strain properties of emergent CWD prions from NO moose and reindeer and to explore the role of residue 226 variation and host CerPrP expression on their transmission and potential for further evolution.

## Results

### Distinct transmission profiles of CWD prions from Norwegian and North American moose are influenced by amino acid variation at PrP residue 226

**Fig 1:**
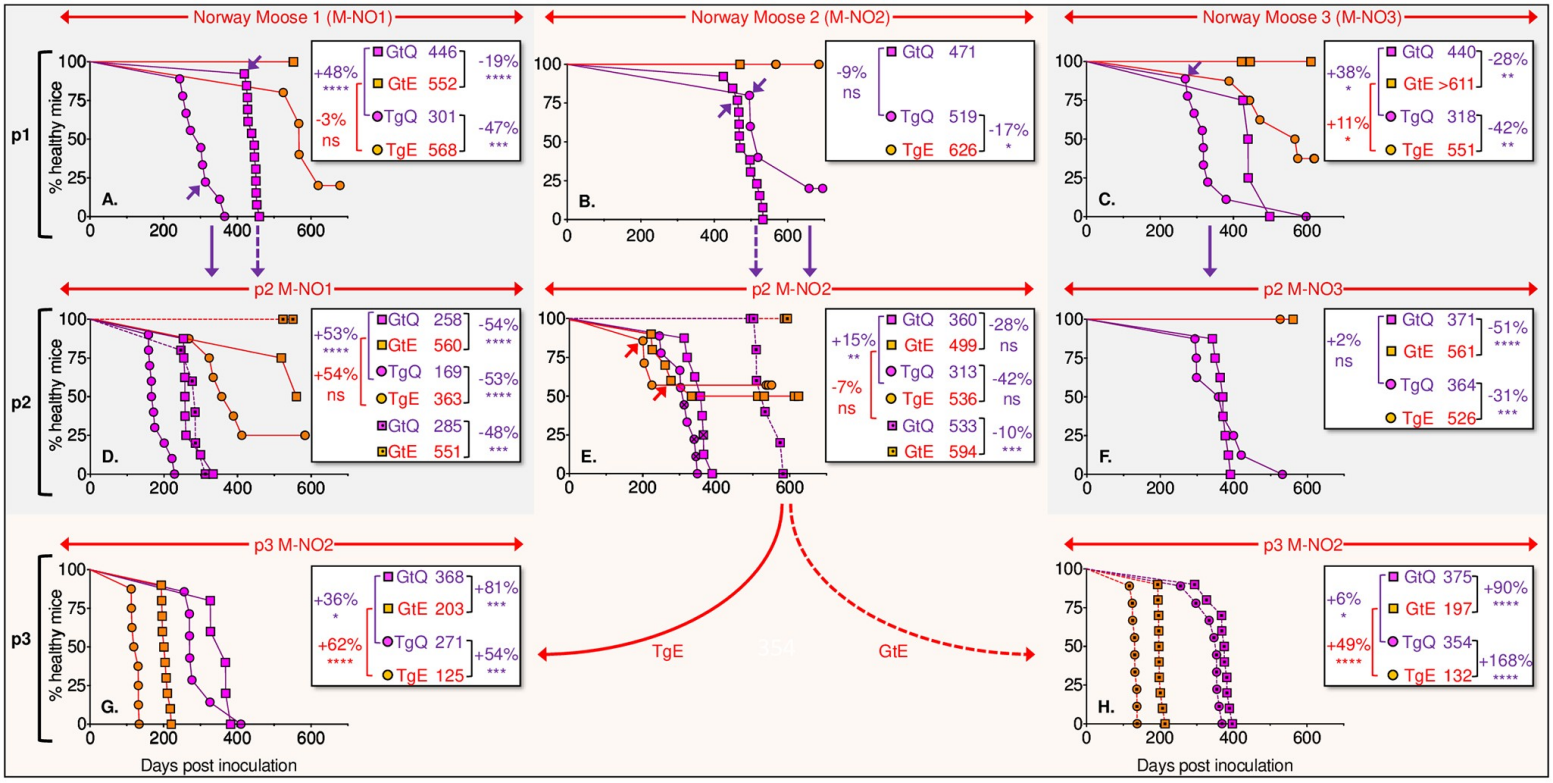
Transmission and adaptation of Norwegian moose CWD prions in mice expressing E226 and Q226 PrP. Survival curves of TgE226 (orange circles), TgQ226 (magenta circles), GtE226 (orange squares), and GtQ226 (magenta squares) following transmission of three CWD isolates from Norwegian moose, referred to as M-NO1, M-NO2, and M-NO3. p1, primary transmissions of prions in brain homogenates from diseased moose; p2 and p3, second and third passages of prions from brain homogenates of diseased mice. **A**., p1 of prions in M-NO1 brain homogenates; **B**., p1 of prions in M-NO2 brain homogenates; **C**. p1 of prions in M-NO3 brain homogenates; **D**., p2 of prions in brain homogenates of TgQ226 or GtQ226 mice infected with M-NO1; **E**., p2 of prions in brain homogenates of TgQ226 or GtQ226 mice infected with M-NO2; **F**., p2 of prions in brain homogenates of TgQ226 mice infected with M-NO-3; **G**., p3 of M-NO2Ad prions in brain homogenates of TgE226 mice; **H**., p3 of M-NO2Ad prions in brain homogenates of GtE226 mice. Mice from which brains were used for serial transmission are indicated by arrows. Dotted squares, Gt mice receiving CWD prions previously passaged in GtQ226 mice. Inserts show median times to disease and statistical differences between different pairs of transmissions indicated by brackets. In **E**., crossed symbols indicate mice with mixed patterns of CNS PrP^Sc^ deposition.

While studies in BV are consistent with differences in the strain properties of prions causing CWD in NO moose, NO reindeer, and NA cervids [44], PrP primary structural mismatches between cervids and BV and the consequent requirement that CWD prions adapt their properties to cause disease in this host presented drawbacks for characterizing emergent NO strains. To circumvent these obstacles we performed experiments in Tg and Gt mice which express CerPrP and therefore lack barriers to CWD prion infection [29,35–37,39]. While TgQ226 and GtQ226 mice express Q226, TgE226 and GtE226 express E226; moose express Q226 [33] (Table 1). Primary transmissions of prions from the CNS of the first three NO moose diagnosed with CWD [23], referred to as M-NO1, M-NO2 and M-NO3 [44], were consistently and significantly more efficient in Tg or Gt mice expressing Q226, producing median times to disease that were 17 to 47% faster than their expression-matched E226 counterparts (Fig 1A–1C and Table 2). While 100% of TgQ226 mice inoculated with M-NO1 and M-NO3 and 80% of TgQ226 mice inoculated with M-NO2 developed disease with median times to onset ranging from ~ 300 to 500 d, transmissions to TgE226 mice either failed to occur (M-NO2) or produced incomplete attack rates with significantly delayed times to onset in those inoculated mice that did develop disease (M-NO1 and M-NO3) (Fig 1A–1C and Table 2). Whereas all GtQ226 mice inoculated with M-NO1, M-NO2 and M-NO3 developed prion disease with median times to onset around 450 d, GtE226 mice remained refractory to disease 550 to 600 d after infection with M-NO1 and M-NO3, and at the time of writing none of nine M-NO2 inoculated GtE226 mice have developed disease after >470 d (Fig 1A and 1C and Table 2). We conclude that Q226 facilitates propagation of NO moose CWD prions while expression of E226 restricts their replication. Since GtQ226 and GtE226 mice express equivalent physiological levels of PrP^C^ and are syngeneic except at codon 226 [39], we further conclude that their differential susceptibility to NO moose CWD prions is the result of amino variation at this position.

**Table 2:** Susceptibility of transgenic and gene-targeted mice expressing Q226 or E226 PrP following intracerebral challenges with North American or Norwegian CWD prions.

**North American moose CWD**							
Inoculum	Tissue	Passage	TgE226	TgQ226	GtE226	GtQ226	Bank vole^1^
M-US1	CNS	p1	286 ± 38 (6/6)	322 ± 28 (8/8)	315 ± 18 (6/6)	414 ± 10 (7/7)	
	CNS	p2	155 ± 21 (9/9)	305 ± 37 (9/9)			
M-CA1	CNS	p1	295 ± 34 (7/7)	412 ± 9 (7/7)	314 ± 13 (5/5)	401 ± 25 (5/5)	311 ± 200 (5/10)
	CNS	p2			208 ± 15 (10/10)	318 ± 39 (9/9)	41 ± 5 (10/10)
	CNS	p3					32 ± 3 (12/12)
M-US2	LRS	p1			306 ± 20 (10/10)	356 ± 44 (6/6)	
M-US3	LRS	p1			210 ± 23 (9/9)	258 ± 72 (8/8)	
**Norwegian moose CWD**							
Inoculum	Tissue	Passage	TgE226	TgQ226	GtE226	GtQ226	Bank vole^1^
M-NO1	CNS	p1	570 ± 39 (4/5)	297 ± 43 (9/9)	553 (0/7)	440 ± 13 (13/13)	318 ± 41 (10/12)
	CNS (TgQ)	p2	337 ± 51 (6/8)	181 ± 27 (10/10)	540 ± 29 (2/5)	271 ± 30 (8/8)	
	CNS (GtQ)	p2			524–551 (0/8)	281 ± 24 (5/5)	
	CNS (BV)	p2					78 ± 4 (10/10)
	CNS (BV)	p3					76 ± 3 (7/7)
M-NO2	CNS	p1	567–685 (0/4)	543 ± 78 (4/5)	470 (0/9)	486 ± 34 (13/13)	459 ± 55 (15/15)
	CNS (TgQ)	p2	210 ± 13 (3/7)	307 ± 38 (9/9)	284 ± 45 (5/10)	352 ± 25 (8/8)	
	CNS (GtQ)	p2			586–594 (0/7)	541 ± 35 (5/7)	
	CNS (BV)	p2					211 ± 18 (8/8)
	CNS (TgE)	p3	123 ± 9 (8/8)	297 ± 55 (7/7)	204 ± 10 (10/10)	354 ± 26 (5/5)	
	CNS (BV)	p3					175 ± 36 (7/7)
	CNS (GtE)	p3	130 ± 7 (9/9)	336 ± 36 (9/9)	199 ± 6 (10/10)	336 ± 31 (10/10)	
M-NO3	CNS	p1	469 ± 81 (5/8)	344 ± 101 (9/9)	422–611 (0/7)	451 ± 33 (4/4)	312 ± 46 (13/13)
	CNS (TgQ)	p2	526 (0/8)	371 ± 81 (8/8)	561 (0/9)	368 ± 17 (8/8)	
**Norwegian reindeer CWD**							
Inoculum	Tissue	Passage	TgE226	TgQ226	GtE226	GtQ226	Bank vole^1^
R-NO1	CNS	p1	590 ± 40 (5/5)	540 (1/5)		508 ± 51 (11/11)	776 (1/7)
	CNS (TgQ)	p2	432 ± 42 (7/7)	365 ± 29 (6/6)	361 ± 35 (7/7)	341 ± 55 (9/9)	
	CNS (GtQ)	p2			371 ± 68 (9/9)	435 ± 63 (7/7)	
R-NO2	CNS	p1	524 ± 51 (3/3)	477 ± 29 (8/8)			690 (0/14)
	CNS (TgQ)	p2	415 ± 29 (8/8)	424 ± 29 (8/8)	359 ± 29 (8/8)	369 ± 37 (7/7)	
	LRS	p1	551 ± 43 (8/8)	598 ± 37 (8/9)			
R-NO3	CNS	p1	497 ± 32 (9/9)	482 ± 12 (6/6)	420 ± 36 (8/8)	430 ± 10 (8/8)	
	LRS	p1	530 ± 26 (8/8)	500 ± 23 (7/7)	437 ± 37 (9/9)	454 ± 14 (4/4)	

Incubation times are expressed as the mean time in days at which inoculated mice first developed ultimately progressive signs of neurological disease ± standard deviation of the mean. Brackets include the numbers of diseased mice/number of inoculated mice.

^1^ Previously reported [29].

**Fig 2:**
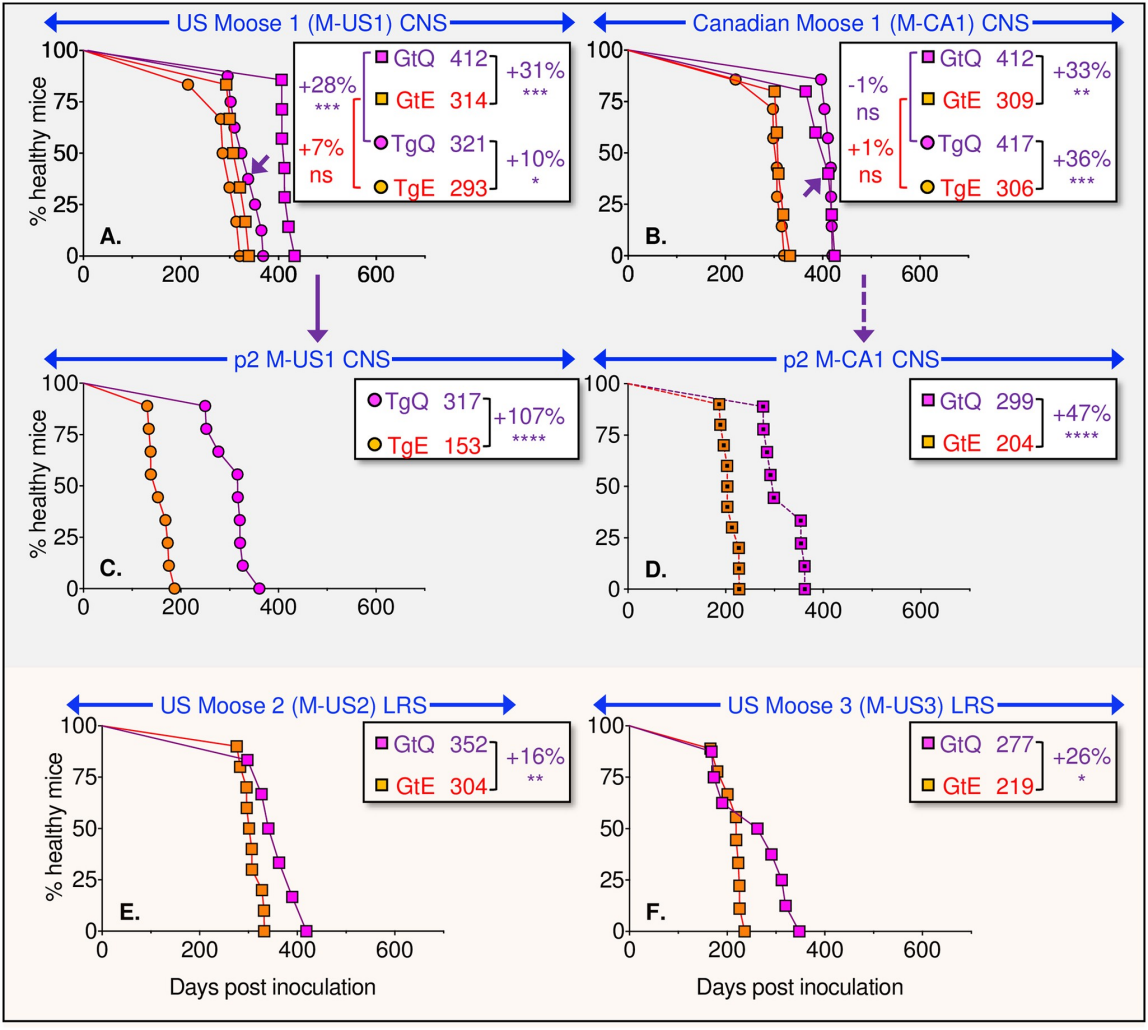
Transmission of North American moose CWD prions to mice expressing E226 and Q226 PrP. Survival curves of TgE226 (orange circles), TgQ226 (magenta circles), GtE226 (orange squares), and GtQ226 mice (magenta squares) following transmission of prions from the CNS or LRS of four different North American moose with CWD, referred to as M-US1, M-CA1, M-US2, and M-US3. **A**., primary transmission (p1) of prions in M-US1 brain homogenates; **B**., p1 of prions in M-CA-1 brain homogenates; **C**., second passage (p2) of prions in brain homogenates of TgQ226 mice infected with M-US1; **D**., p2 of prions in brain homogenates of GtQ226 mice infected with M-CA1; **E**., p1 of prions in M-US2 lymphoid tissue homogenates; **F**., p1 of prions in M-US3 lymphoid tissue homogenates. Mice from which brains were used for serial transmission are indicated by arrows. Dotted squares, Gt mice receiving CWD prions previously passaged in GtQ226 mice. Inserts show median times to disease and statistical differences between different pairs of transmissions indicated by brackets.

Our findings with NO moose CWD stand in contrast to our previous analyses of NA deer and elk CWD prions which, while causing disease in E226- as well as Q226-expressing mice, did so more rapidly in the E226 background [39]. To distinguish whether this discrepancy resulted either from general idiosyncratic features of moose prions or from specific differences in the strain properties of NA and NO CWD, we assessed transmission of four NA moose CWD isolates referred to as M-US1, M-US2, M-US3, and M-CA1. In contrast to NO moose CWD and in accordance with the properties of NA deer and elk CWD prions [39], primary transmissions of NA moose CWD elicited disease with 100% attack rates in Tg and Gt mice, producing median times to clinical onset that were 10 to 33% faster in mice expressing E226 compared to their expression-matched Q226 counterparts (Fig 2A, 2B, 2E and 2F and Table 2). Our comparisons of NO and NA CWD are in accordance with previous findings which concluded that variation at residue 226 controls the selection and propagation of CWD prion strains [39]. We further conclude that whereas the overlapping effects of PrP residue 226 variation on the propagation of NA moose, deer, and elk CWD prions are consistent with infection by strains with shared properties, the distinct responses of NO moose CWD prions to variation at residue 226 indicates that the strain characteristics of these emergent CWD prions are different from those of established NA CWD.

The divergent transmission profiles of NO moose and NA CWD observed on primary passage were recapitulated during iterative passages in Tg and Gt mice. While all TgQ226 and GtQ226 mice inoculated with M-NO1 and M-NO3 prions previously passaged in TgQ226 and GtQ226 mice succumbed to disease, similarly challenged TgE226 and GtE226 mice were either completely resistant or else disease was only registered in subsets of inoculated mice. As a result, median incubation times of serially passaged NO moose CWD were 24 to 54% longer in E226 mice than in their Q226 counterparts (*P* ≤ 0.001 to 0.0001) (Fig 1D and 1F and Table 2). Similar results were observed following transmission of M-NO2 CWD from diseased GtQ226 mice (Fig 1E and Table 2). By contrast, sub-passage of NA CWD M-US1 and M-CA1 prions from TgQ226 and GtQ226 mice produced disease upon transmission to E226 as well as Q226 mice with significantly prolonged onsets in Q226 backgrounds (Fig 2C and 2D and Table 2). These findings provide further support for our conclusions that residue 226 influences the propagation of distinct NO moose and NA CWD prion strains, that transmission of NO moose CWD prions is favored by expression of Q226 and relatively restricted by E226, and that replication of NA CWD prions is facilitated by both amino acid variants but favored by E226.

### Adaptation of Norwegian moose CWD prions

Primary transmission of M-NO2 CWD caused disease in TgQ226 and GtQ226 but not TgE226 or, at the time of writing (>470 d) GtE226 mice (Fig 1B and Table 2). While secondary passage also produced disease in all inoculated Q226 mice and the majority of E226 mice remained free of disease, subsets of TgE226 (3/7) and GtE226 mice (5/10) developed disease with incubation times that were more rapid than their Q226-expressing counterparts (Fig 1E and Table 2). To characterize the properties of prions causing unusually rapid disease in normally resistant E226 mice we performed further iterative passages. Remarkably, the transmission profile of these prions was distinct from the original M-NO2 prions. Upon tertiary passage, all inoculated Q226 and E226 mice developed disease with onsets that were more rapid in E226 mice than their Q226 counterparts (Fig 1G and 1H and Table 2). Our findings are consistent with evolution of M-NO2 moose CWD prions during iterative passage to produce a strain with transmission properties reminiscent of established NA CWD. We refer to these adapted prions as M-NO2Ad.

### The transmission properties of Norwegian reindeer CWD are distinct from Norwegian moose and NA CWD

**Fig 3:**
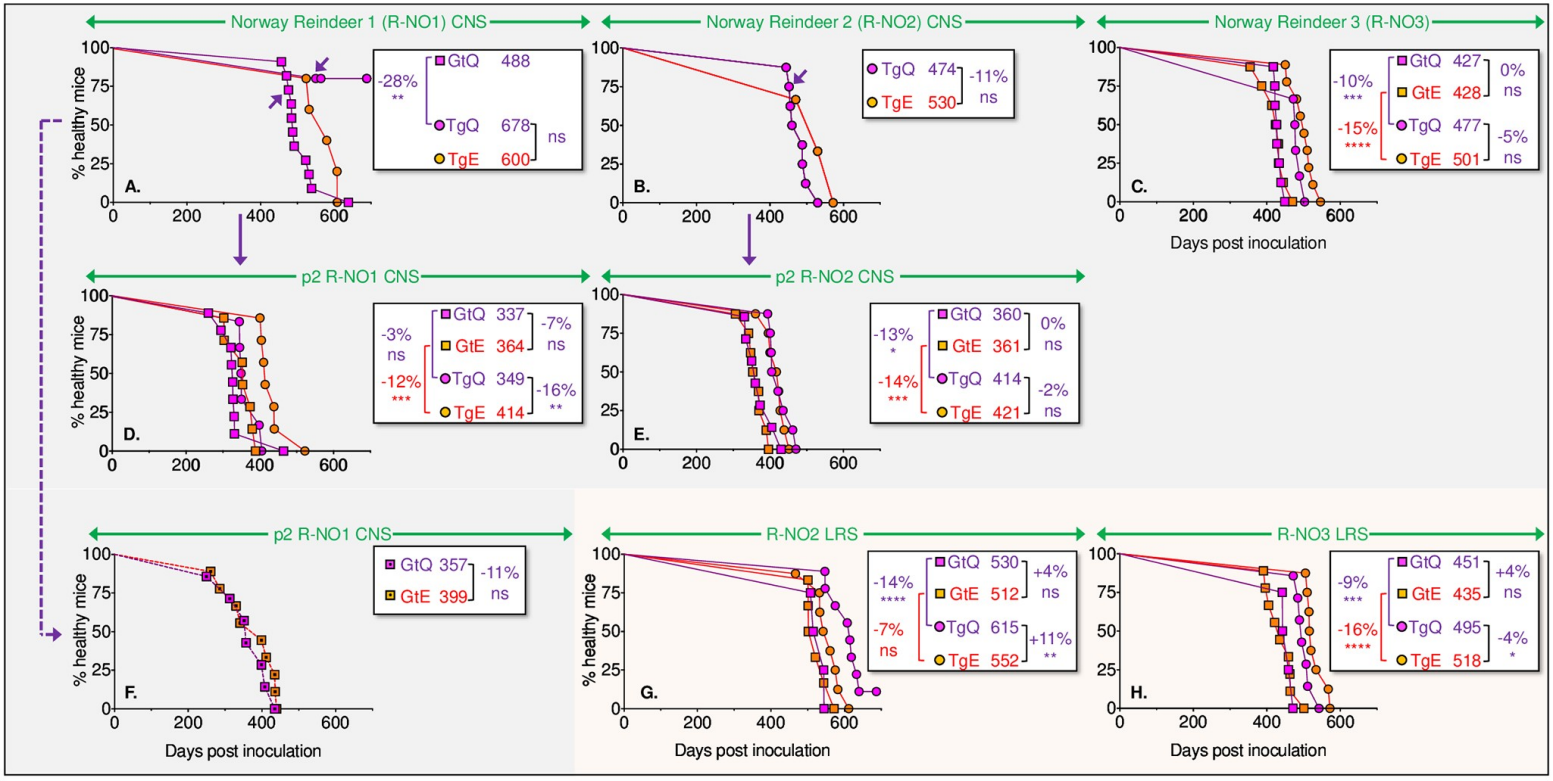
Transmission of CWD prions from Norwegian reindeer to mice expressing E226 and Q226 PrP. Survival curves of TgE226 (orange circles), TgQ226 (magenta circles), GtE226 (orange squares), and GtQ226 mice (magenta squares) following transmission of CWD prions from the CNS or LRS of three Norwegian reindeer CWD isolates, referred to as R-NO1, R-NO2, and R-NO3. **A**., primary transmission (p1) of prions in R-NO1 brain homogenates; **B**., p1 of prions in R-NO2 brain homogenates; **C**., p1 of prions in R-NO3 brain homogenates; **D**., second passage (p2) of prions in brain homogenates of diseased TgQ226 mice infected with R-NO1; **E**., p2 of prions in brain homogenates of diseased TgQ226 mice infected with R-NO2; **F**., p2 of prions in brain homogenates of diseased GtQ226 mice infected with R-NO1; **G**., p1 of prions in R-NO2 lymphoid tissue homogenates; **H**., p1 of prions in R-NO3 lymphoid tissue homogenates. Mice from which brains were used for serial transmissions are indicated by arrows. Dotted squares, Gt mice receiving CWD prions previously passaged in GtQ226 mice. Inserts show median times to disease and statistical differences between different pairs of transmissions indicated by brackets.

We challenged TgQ226, TgE226, GtQ226 and GtE226 mice with the three initial cases of CWD detected in NO reindeer, referred to as R-NO1, R-NO2, and R-NO3 [21,22,44]. Reindeer express Q226 [34] (Table 1). In contrast to the resistance of BV during primary transmission of NO reindeer isolates R-NO1 and R-NO2 [44] (Table 2) and with the exception of incomplete transmission of R-NO1 to TgQ226, Tg and Gt mice were uniformly susceptible upon primary passage of NO reindeer CWD prions. In addition, we recorded no significant differences in times to onset when we compared disease outcomes in Q226 with E226 mice (Fig 3A–3C and Table 2). Incubation times generally stabilized around 330 to 420 d upon sub passage of R-NO1 and R-NO2 from diseased Q226 mice and, with the exception of R-NO1 transmissions to Tg mice (Fig 3A and Table 2), we continued to observe no significant differences in times to CWD onset between Q226 and E226 mice (Fig 3D, 3E and 3F and Table 2).

The presence of PrP^Sc^ in lymphoid tissues of NO reindeer [21] afforded additional opportunities to assess the transmission properties of NO reindeer CWD prions and to build upon our previous comparative analyses of LRS- and CNS-resident CWD prions [37]. While we observed no differences in R-NO2(LRS) and R-NO3(LRS) prion incubation times in GtQ226 and GtE226 mice (Fig 3G and 3H and Table 2), M-US2(LRS) and M-US3(LRS) produced significantly faster disease onsets in GtE226 compared to GtQ226 mice (Fig 2E and 2F and Table 2). Transmissions to Tg mice produced a less consistent picture with R-NO2(LRS) and R-NO3(LRS) prions producing faster disease in TgE226 and TgQ226 respectively (Fig 3G and 3H and Table 2). Collectively our findings show that while BV are relatively resistant upon primary transmission of NO reindeer CWD [44] (Table 2), transmission to Gt and Tg mice occurred with high efficiency. Additionally, the effects of primary structural differences at residue 226 on prion propagation were less pronounced for NO reindeer CWD compared to either NO moose or NA CWD. We conclude that the strain properties of prions causing NO reindeer CWD are distinct from either those causing disease in NO moose or NA CWD.

In summary, our studies of NO and NA CWD prions in mice expressing Q226 and E226 reveal the following distinct patterns: 1) Transmission of US or Canadian moose, deer or elk CWD prions occurred in both backgrounds but was facilitated by expression of E226 [39] (Fig 2 and Table 2); 2) Susceptibility to NO reindeer CWD prions was relatively unaffected by amino acid variation at residue 226 (Fig 3 and Table 2); 3) Transmission of NO moose CWD prions was favored in mice expressing Q226 and restricted by E226 (Fig 1 and Table 2); (4) Iterative passage of NO moose isolate M-NO2 resulted in selection of a kinetically optimized derivative in E226 mice referred to as M-NO2Ad, subsequent transmissions of which revealed characteristics akin to NA CWD (Fig 1G and 1H and Table 2).

### In vitro prion amplification recapitulates the effects of amino acid variation at PrP residue 226 on NO and NA prion conversion

**Fig 4:**
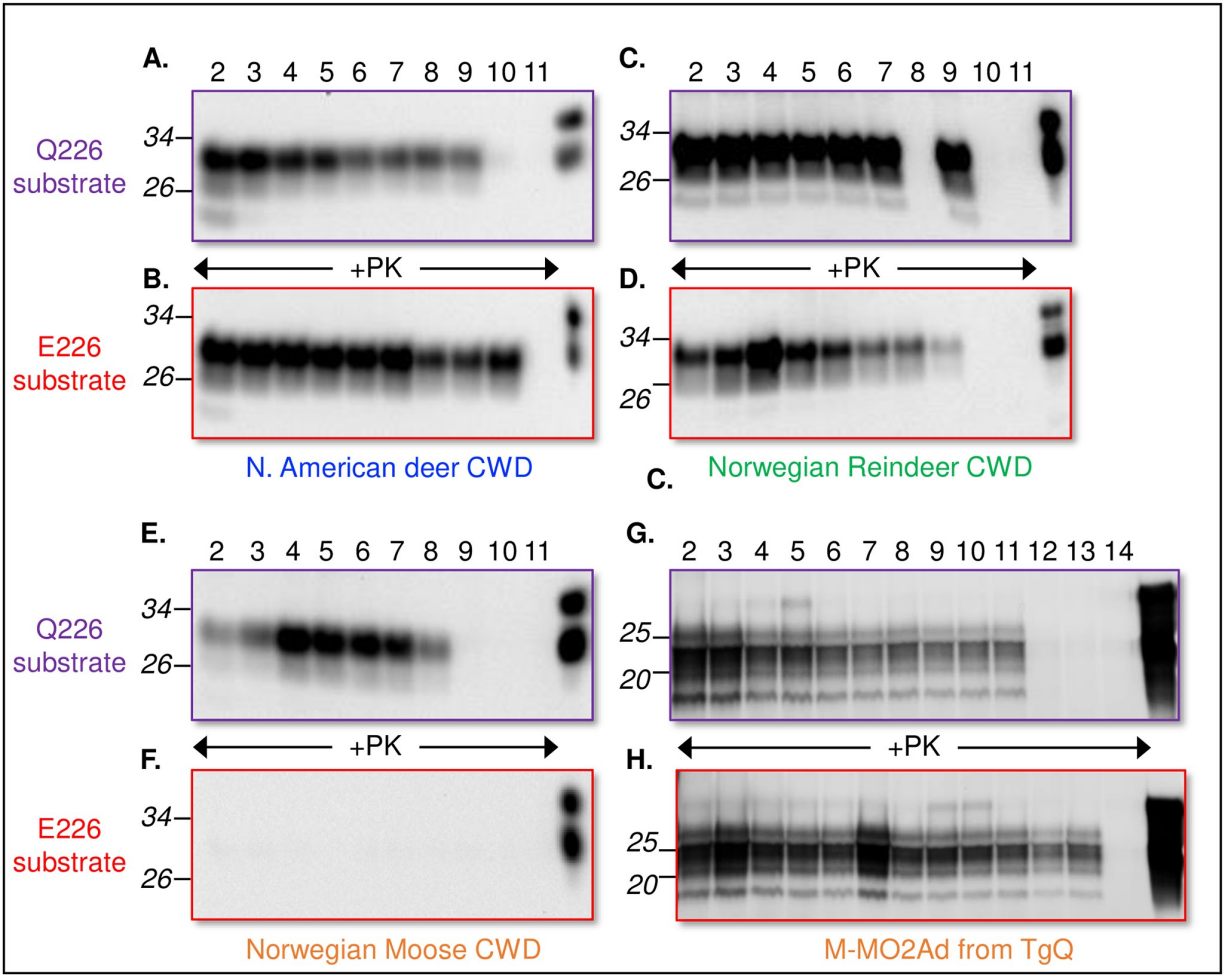
In vitro conversion properties of North American, Norwegian, and adapted Norwegian moose CWD prions using Q226 and E226 substrates. Homogenized CWD-infected brain materials used to initiate the PMCA seeding reaction is considered as 10^−2^ in the dilution series. This material was serially diluted in 10-fold increments up to 10^−14^ in uninfected brain homogenates from TgQ226 and TgE226 mice, represented as number series above immunoblots. Reactions were digested with PK as indicated. Undigested normal brain homogenates of TgE226 and TgQ226 mice are included in the final lane of each immunoblot. The positions of molecular weight markers are indicated.

Previous studies revealed that aspects of the transmission properties of CWD prions were broadly recapitulated in vitro during protein misfolding cyclic amplification (PMCA) [36,45]. We therefore used brain extracts from Tg mice to assess whether NO and NA CWD prions differed in their amplification properties when E226 and Q226 were supplied as templates for conversion to PrP^Sc^. Whereas E226 and Q226 substrates both sustained the conversion of NA CWD (Fig 4A and 4B) and NO reindeer CWD (Fig 4C and 4D), amplification of NO moose CWD occurred in the presence of Q226 (Fig 4E) but not when E226 was supplied as substrate (Fig 4F). Since transmission of M-NO2Ad prions produced susceptibilities and disease kinetics in Q226 and E226 mice that were concordant with those of NA CWD (Fig 1G and 1H and Table 2), we also asked whether the PMCA potential of M-NO2 evolved following its transition to M-NO2Ad. M-NO2Ad prions isolated from the brains of TgQ226 mice after three iterative passages of M-NO2 (Fig 1G) promoted efficient amplification of both E226 and Q226 substrates during PMCA (Fig 4G and 4H). These in vitro results support our findings from transmission studies and collectively indicate that while NO moose CWD prions propagate only when Q226 is supplied as template for prion conversion, adaptation of M-NO2 results in M-NO2Ad prions which resemble NA CWD in their capacity to amplify both E226 and Q226 substrates.

### Unexpected responses of gene-targeted mice and their overexpressing transgenic counterparts to CWD prions

The availability of Gt mice and their Tg counterparts on an identical inbred FVB background enabled us to compare the effects of physiological PrP expression with CNS overexpression on susceptibility to CWD prions. In accordance with the longstanding view that the kinetics of prion disease onset are inversely proportional to levels of PrP expression in the CNS [46,47], our previous studies showed that intracerebral (ic) inoculation with NA CWD prions produced disease more rapidly in Tg mice compared to their Gt mouse counterparts expressing physiologically accurate but lower levels of PrP [39]. Accordingly, whereas the 7 to 53% faster median times to disease onset of M-NO1 and M-NO3 moose CWD in overexpressing Tg mice compared to their Gt counterparts was anticipated (Fig 1A and 1C and Table 2), the more efficient transmission of M-NO2 in GtQ226 compared to TgQ226 mice was unexpected (Fig 1B and Table 2). Our surprise was more pronounced when we assessed the transmission properties of NO reindeer CWD prions during primary and secondary passages in Gt and Tg mice. Here, transmission was either equally efficient or more frequently faster in Gt mice compared to their overexpressing, residue 226-matched Tg counterparts (Fig 3). Our findings are consistent with the notion that the transmission efficiency of particular prion strains is not governed solely by levels of PrP expression in the CNS and that physiologically accurate PrP expression in peripheral compartments is at least one additional parameter that facilitates this process.

### Distinct patterns of PrP^Sc^ deposition in brains of mice infected with NO moose, NO reindeer, and NA CWD

**Fig 5:**
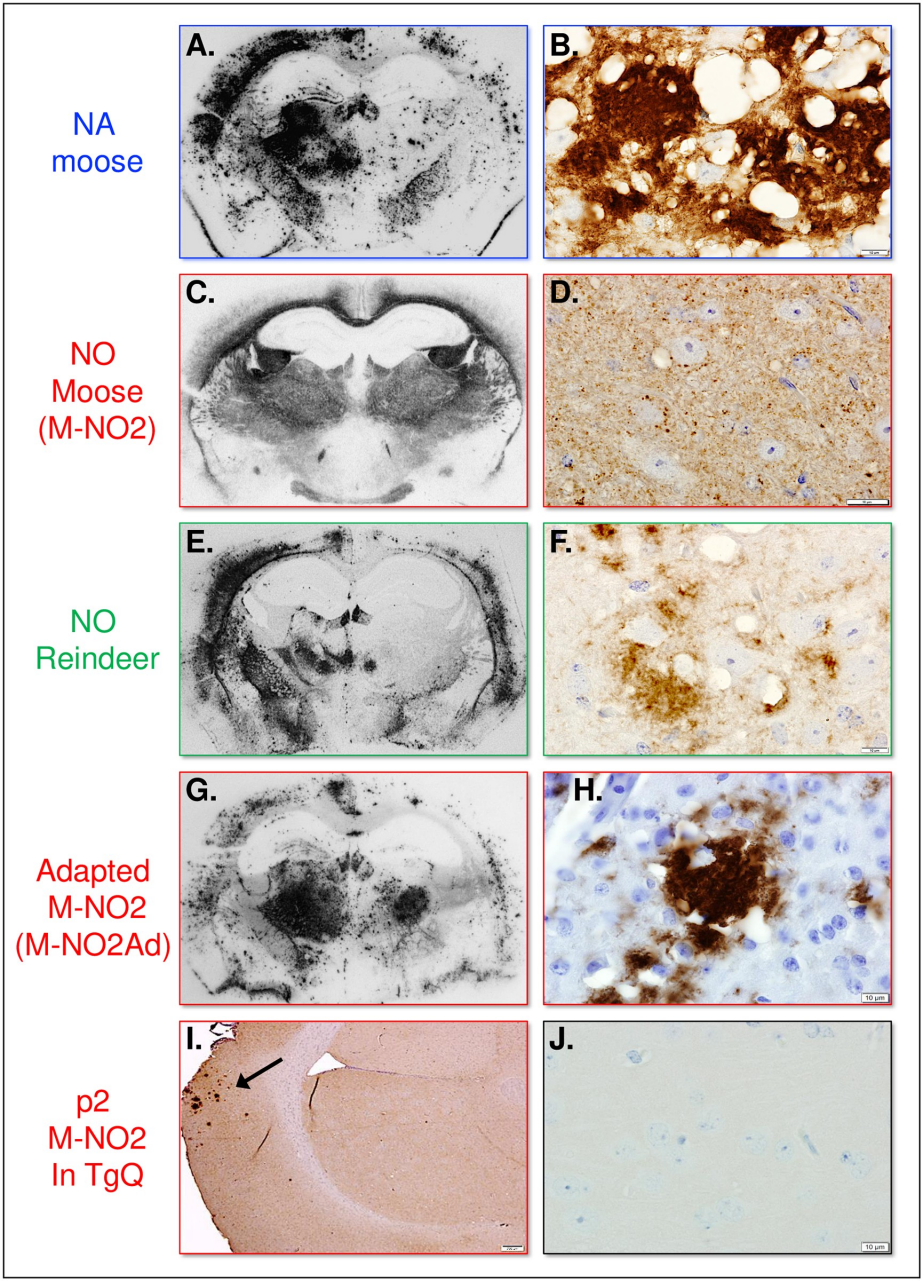
Analysis of PrP^Sc^ in the CNS of CWD infected Tg and Gt mice by histoblotting and immunohistochemistry. Histoblotted coronal brain sections at the level of the hippocampus and thalamus (**A**., **C**., **E**., **G**.,) and immunohistochemical (IHC) analysis (**B**., **D**., **E**., **F**., **H**.,) of PrP^Sc^ deposition in the region of the hippocampus of Q226 mice infected with North American moose CWD (**A**., **B**.); Norwegian moose isolate M-NO2 (**C**., **D**.); Norwegian reindeer (**E**., **F**.); and adapted Norwegian moose CWD, M-NO2Ad (**G**., **H**.). Arrow in **I**. indicates location of M-NO2Ad PrP^Sc^ plaque aggregates in the cortex at the level of the hippocampus during second passage of M-NO2 in TgQ226 mice. **J**., section from the region of the hippocampus of uninfected TgQ226 mice. PK treated histoblots were probed with mAb PRC5. IHC sections were probed with Fab D18. Bar in **B**., **D**., **F**., **H**., **J**. = 10 μM; bar in **I**. = 200 μM.

To corroborate our proposal that the distinct transmission properties of NO and NA CWD prions reflect prion strain differences, we used histoblotting [48] and immunohistochemical (IHC) analyses to compare the distributions and morphological properties of PrP^Sc^ in the CNS of diseased Q226 mice. Consistent with our previous findings with NA deer and elk CWD [38,39], NA moose CWD prions produced intensely staining and highly compacted PrP^Sc^ aggregates which frequently coalesced into large, disorganized amalgamations (Fig 5A and 5B). These depositions were asymmetrically distributed in the cortex, hippocampus, thalamus, and hypothalamus of diseased mice ipsilateral to the hemisphere of inoculation (Fig 5A).

PrP^Sc^ labeling in the CNS of Q226 mice infected with NO moose CWD (Fig 5C and 5D) was clearly different from Q226 mice infected with NA CWD (Fig 5A and 5B). Instead of asymmetrical plaque deposits, the pattern of PrP^Sc^ distribution was widespread, diffuse, and consistently symmetrical throughout the CNS (Fig 5C). IHC analysis confirmed that PrP^Sc^ accumulated as diffuse and/or minuscule punctate PrP^Sc^ deposits reminiscent of the staining pattern reported in brain sections of CWD-affected NO moose [23] (Fig 5D).

IHC analyses of TgQ226 and GtQ226 mice infected with M-NO2 CWD prions at passage 2 (Fig 1E) revealed multiple examples of brain sections that contained infrequent and randomly dispersed groups of PrP^Sc^ plaques in a background of diffuse staining (arrow in Fig 5I). We infer that these PrP^Sc^ plaques represent the emergence of M-NO2Ad, while diffuse PrP^Sc^ corresponds to M-NO2. IHC analyses of M-NO2Ad PrP^Sc^ in the brains of Q226 mice at passage 3 revealed that the distribution and morphology of PrP^Sc^ was exclusively asymmetrical and aggregated (Fig 5G and 5H), was wholly distinct from the diffuse pattern produced by M-NO2 at passage 1 (Fig 5C and 5D), and was very similar to the PrP^Sc^ plaque aggregates associated with infection of Q226 mice NA moose CWD (Fig 5A and 5B) or NA deer and elk CWD [39]. These results provide further evidence that the properties of M-NO2Ad correspond to those of NA CWD.

The morphology and distribution of PrP^Sc^ in brain sections of mice infected with NO reindeer CWD prions were different from and intermediate between those of PrP^Sc^ produced by NO or NA moose CWD. Although reindeer CWD PrP^Sc^ also accumulated in asymmetrically distributed aggregates in the CNS of Q226 mice (Fig 5G), they were generally less densely compacted (Fig 5F) than the highly coalescent aggregates associated with NA CWD (Fig 5B).

### Comparative analysis of PrP^Sc^ constituting North American and Norwegian CWD prions

We assessed proteinase K (PK)-resistant PrP^Sc^ in the brains of CWD-affected Tg and Gt mice by western blotting. PrP^Sc^ levels in the CNS of NO moose were ~ 10-fold lower than PrP^Sc^ levels in brain extracts of NA cervids (S1A Fig). Following passage of M-NO1 and M-NO2 to TgQ226 and GtQ226 mice PrP^Sc^ levels were respectively ~ 15% and ~ 20% of PrP^Sc^ levels produced in the brains of mice infected with NA moose CWD (*P* ≤ 0.0001 in both cases) (S1C Fig). Since differences in the levels of CNS PrP^Sc^ accumulation were sustained upon transmission of NO moose and NA CWD to TgQ226 and GtQ226 mice (S1B Fig) we conclude that they reflect inherent differences in the strain properties of these prions.

We also found that electrophoretic migration patterns of PK-resistant PrP^Sc^ fragments produced in mice infected with M-NO1 were different from those found in mice infected with M-NO2 or M-NO3. Whereas M-NO1 produced a single non-glycosylated PrP^Sc^ fragment, M-NO2 and M-NO3 produced an additional non-glycosylated PK-resistant species. The faster migrating fragment in this doublet was less abundant than its higher molecular weight (MW) counterpart and was revealed to greater effect when samples were overloaded on western blots (red arrows in S1D Fig). These differences in the patterns of M-NO2 and M-NO3 PrP^Sc^ compared to M-NO1 PrP^Sc^ upon transmission to Q226 mice appear to correspond to originally observed differences in brain samples of infected NO moose [23].

**Fig 6:**
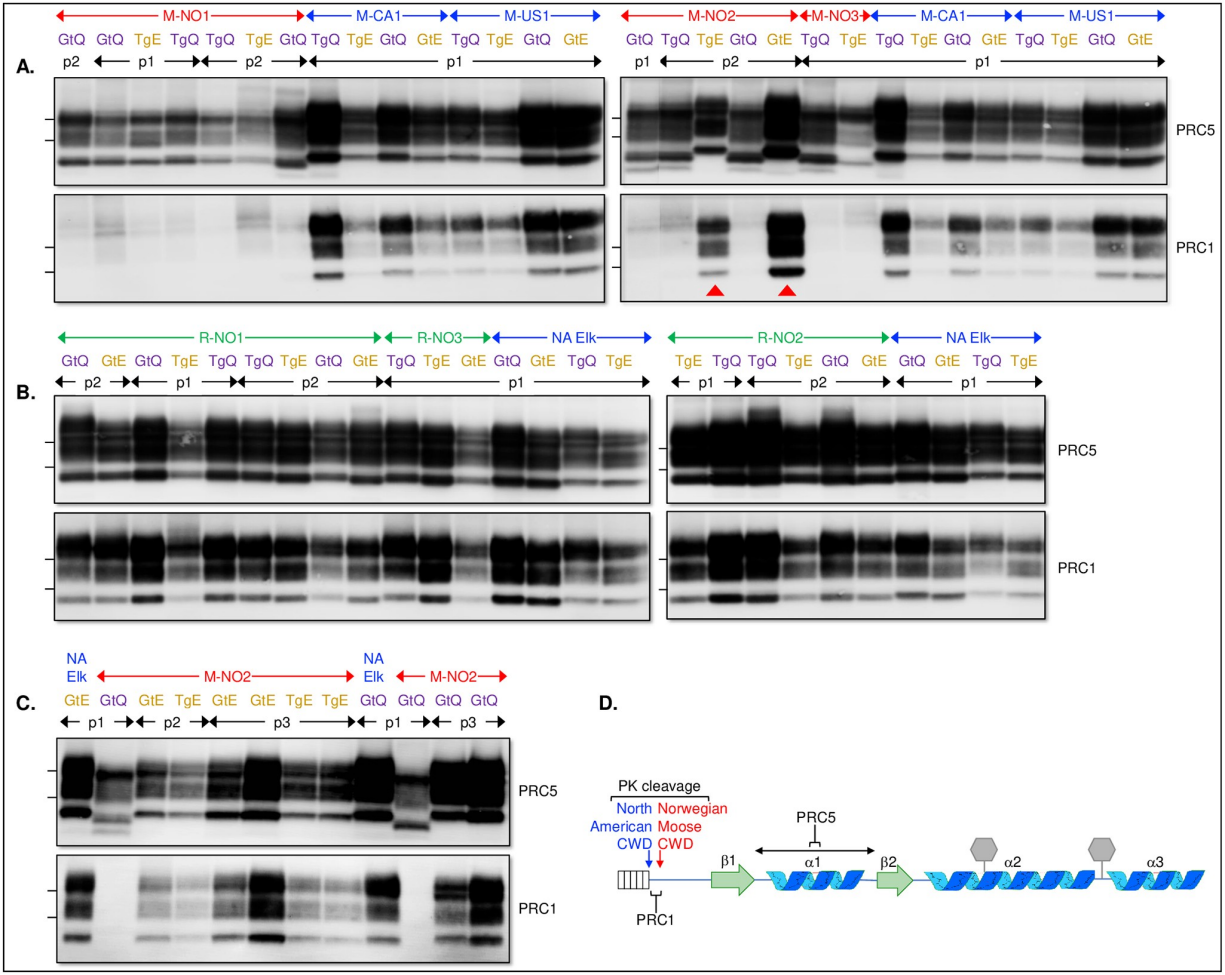
Immunoblot analysis of PrP^Sc^ in the CNS of CWD infected mice. Western blot analysis of PrP^Sc^ produced in the CNS of mice infected with **A**. North American moose CWD (M-CA1, M-US1) or Norwegian moose CWD (M-NO1, M-NO2, M-NO3) at various passages (p1 and p2). **B**. North American elk CWD or Norwegian reindeer CWD (R-NO1, R-NO2, R-NO3) at various passages (p1 and p2). **C**. Analysis of PrP^Sc^ during adaptation of M-NO2 to form M-NO2Ad during various passages (p1, p2, p3). Immunoblots were probed with either mAb PRC5 or PRC1 as indicated. **D**. Location of PrP epitopes for mAbs PRC1 and PRC5. The position of molecular weight markers approximating 25- and 20-kDa are shown to the left of blots.

We made further assessments of PrP^Sc^ in the CNS of Q226 mice by western blotting using monoclonal antibodies (mAbs) directed against different regions of PrP. While mAb PRC5 recognizes an epitope that includes residues 135 and 162 in the structured globular domain, the epitope of mAb PRC1 encompasses Q at residue 95 immediately distal to the site of PK cleavage of NA PrP^Sc^ [49] (Fig 6D). Western blotting with mAb PRC5 showed that the electrophoretic migration of NO moose PrP^Sc^ fragments was more rapid than NA PrP^Sc^ (Fig 6A). In addition, NO moose PrP^Sc^ was refractory to detection by mAb PRC1 confirming that its lower apparent MW was a consequence of PK cleavage at a more C-terminal site than in NA PrP^Sc^. These findings agree with western blotting analyses of PrP^Sc^ in the CNS of CWD-affected NO moose using similar discriminatory antibodies [23]. Our findings provide molecular confirmation of strain differences between PrP^Sc^ constituting NO moose and NA CWD and demonstrate that Tg and Gt mice recapitulate the features of NO moose PrP^Sc^ produced in the natural host.

We also used western blotting with discriminatory mAbs to assess whether the properties of PrP^Sc^ changed during evolution of M-NO2 to M-NO2Ad. Second passage of prions in TgE226 and GtE226 mice that developed disease with rapid incubation times after challenges with M-NO2 (Fig 1E) produced PrP^Sc^ with slower electrophoretic migration properties than PrP^Sc^ generated after passage 1 of M-NO2, and this slower migrating PrP^Sc^ was also detected by mAb PRC1 (red arrowheads in Fig 6A). Further passage (p3) also produced slower migrating, PRC1-reactive PrP^Sc^ in the brains of all analyzed Q226 and E226 mice (Fig 6C). These findings provide further confirmation that M-NO2 evolved during passaging to produce an adapted M-NO2Ad derivative comprised of PrP^Sc^ with properties resembling NA CWD.

Western blotting of PrP^Sc^ produced during primary or secondary passages of NO reindeer CWD in Tg or Gt mice revealed no differences in apparent MW or reactivity with mAb PRC1 when compared to NA CWD (Fig 6B). While these findings are in agreement with western blotting results of PrP^Sc^ in the CNS of CWD-affected NO reindeer [21], they differ from the results of studies in BV where the apparent MW of PrP^Sc^ in the CNS of the single diseased R-NO1 infected BV was different from that of PrP^Sc^ produced in response to infection with NA CWD prions [44]. We conclude that Tg and Gt mice reproduce the features of NO reindeer PrP^Sc^ produced in the natural host.

### Gene targeted mice recapitulate the distinctive lymphotropic properties of Norwegian reindeer and moose CWD and reveal acquisition of lymphotropism during adaptation of M-NO2 to M-NO2Ad

**Fig 7:**
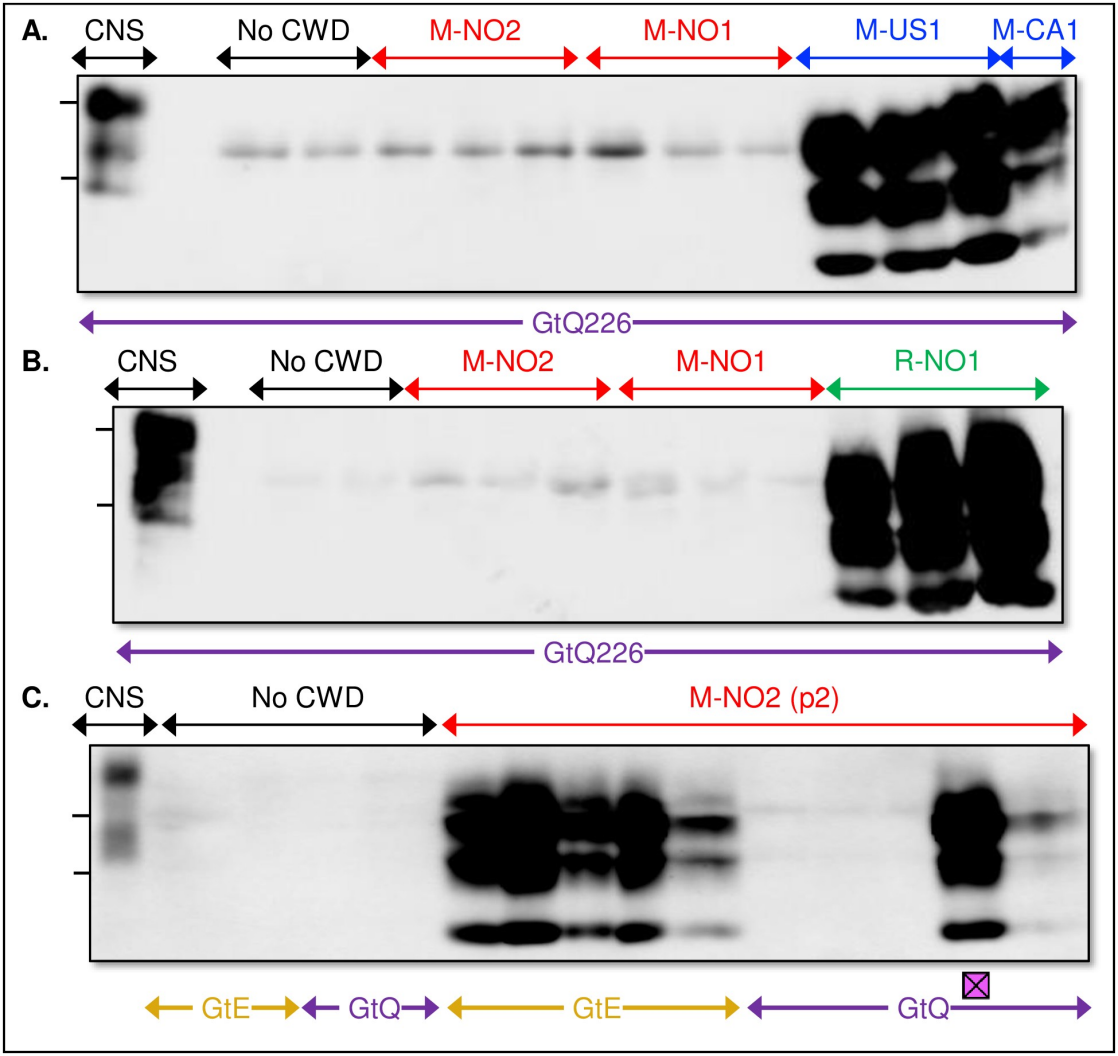
Strain dependent PrP^Sc^ accumulation in spleens of CWD infected GtQ226 mice. Immunoblots showing PrP^Sc^ in the spleens of GtQ226 mice following challenge with **A**., North American moose CWD isolates M-US1 and M-CA1, but not in spleens of GtQ226 mice challenged with Norwegian moose CWD isolates M-NO1 and M-NO2; and **B**., Norwegian reindeer CWD isolate R-NO1. **C**. Accumulation of PrP^Sc^ in the spleens of mice propagating adapted M-NO2Ad Norwegian moose prions. Crossed squares, GtQ226 mouse with mixed patterns of aggregated and diffuse CNS PrP^Sc^ deposition following p2 of M-NO2 in Fig 1E. Spleen homogenates (1 mg) were treated with 50 μg/ml PK. Membranes were probed with mAb PRC5. All samples were treated with PK except in lane 1 of each immunoblot to show PrP^C^ in the CNS of GtQ226 mice. No CWD, spleen preparations from uninfected GtQ226 mice. CNS, undigested PrP from the CNS of Gt mice. The positions of molecular weight markers corresponding to 34- and 27-kDa are shown.

Analyses of diseased NO cervids showed that PrP^Sc^ was detected only in lymphoid tissues of CWD-affected NO reindeer and not in the LRS of CWD-affected NO moose [21,23]. Our previous studies showed that while TgQ226 mice were relatively resistant to intraperitoneal (ip) CWD challenges and failed to accumulate PrP^Sc^ in their spleens, this was not the case in GtQ226 mice which were equally susceptible to ip and ic challenges and accumulated splenic PrP^Sc^ [39]. We therefore asked whether GtQ226 mice would recapitulate the different lymphotropic properties of NO moose and reindeer CWD prions revealed during analyses of their naturally-affected hosts [21,23]. Western blotting analyses showed that PrP^Sc^ accumulated in the spleens of GtQ226 mice infected with NA moose CWD (Fig 7A) and NO reindeer CWD (Fig 7B) but not in the spleens of GtQ226 mice infected with NO moose CWD (Fig 7A and 7B). We therefore conclude that differences in lymphoid PrP^Sc^ accumulation in NO moose and reindeer are the result of inherent lymphotropic strain differences between NO moose and reindeer CWD prions that are recapitulated upon transmission to GtQ226 mice.

The ability to address lymphotropic strain properties of CWD in Gt mice allowed us to ask whether strain switching of non-lymphotropic M-NO2 resulted in the coincident acquisition of lymphotropism by M-NO2Ad. Western blotting of spleen extracts from all five GtE226 mice that developed prion disease at times earlier than their GtQ226 counterparts upon second passage of M-NO2 (Fig 1E) showed that they contained levels of PrP^Sc^ comparable (Fig 7C) to those found in the spleens of Gt mice infected with lymphotropic NA and NO reindeer CWD strains (Fig 7A and 7B). Similar analysis of the five diseased GtQ226 mice following second passage of M-NO2 showed that PrP^Sc^ was present at high levels in the spleen of the single mouse in which IHC analysis of CNS sections revealed M-NO2Ad PrP^Sc^-containing plaques (Figs 1E, 7C and 5I), while a relatively low level of PrP^Sc^ was detected in the spleen of only one of the four remaining mice in which IHC analyses detected only diffuse CNS PrP^Sc^ deposition characteristic of M-NO2 (Figs 1E, 7C and 5D). Our results provide support for the proposal that adaptation of M-NO2 CWD resulted in concomitant acquisition of lymphotropic strain properties by the resulting M-NO2Ad prions.

### Distinct conformational properties of NA and NO CWD prions and adapted derivatives

**Fig 8:**
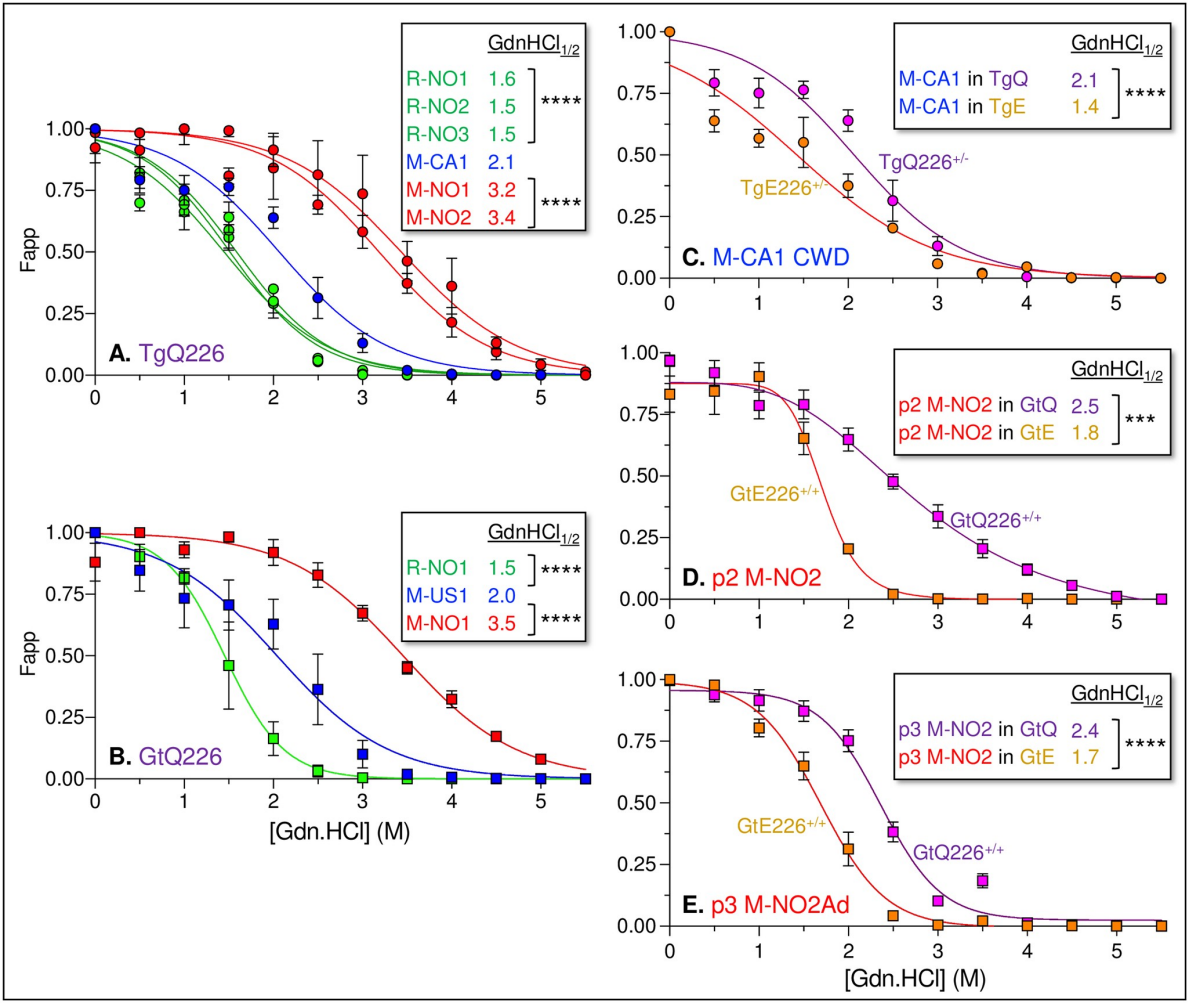
Conformational stabilities of North American CWD, Norwegian CWD, and adapted Norwegian CWD. Responses of CWD prions to denaturation with increasing concentrations of GdnHCl. The percentage of PK-resistant PrP^Sc^ was plotted as a function of GdnHCl concentration (M). Sigmoidal dose-response curves were plotted using a four-parameter algorithm and nonlinear least-square fit. F_app_, fraction of apparent PrP^Sc^ = (maximum signal–individual signal)/(maximum signal–minimum signal). Error bars, ± SEM of data from analyses of three animals per group. GdnHCl_1/2_ values (M) of PrP^Sc^ from each infection are reported to the right-hand side of each graph. **** and ***, *P* ≤ 0.0001 and *P* ≤ 0.001 differences in GdnHCl_1/2_ values between M-CA1 and NO moose or reindeer CWD isolates in **A**., and between M-US1 and NO moose and reindeer CWD isolates in **B**., and paired analyses in **C**.–**D**. calculated by pairwise analysis of GdnHCl_1/2_ values from best fit curves. Brain extracts from (**A**.) diseased TgQ226 mice; (**B**.) diseased GtQ226 mice. **C**., conformational stability analysis of North American moose CWD in GtE226 and GtQ226 mice. **D.–E**. Analysis of conformational adaptation during transition of M-NO2 CWD prions to M-NO2Ad.

We assessed strain-dependent differences in prion conformational stability by measuring responses of NO moose CWD, NO reindeer CWD, and NA CWD PrP^Sc^ to treatment with increasing amounts of denaturant [50] (Fig 8). The concentration of guanidine hydrochloride (GdnHCl) producing half-maximal denaturation of PK-resistant PrP^Sc^ (GdnHCl_1/2_) in TgQ226 mice infected with the three NO reindeer CWD isolates clustered in the range of 1.5 to 1.6 M (Fig 8A). In contrast, the right-shifted GdnHCl denaturation profiles of PrP^Sc^ in the brains of TgQ226 mice infected with M-NO1 and M-NO2 and their 3.2 to 3.4 M GdnHCl_1/2_ values indicated that the stabilities of these NO moose CWD prion conformers were distinct from and greater than those of NO reindeer CWD prions (*P* ≤ 0.0001) (Fig 8A). The denaturation profile of PrP^Sc^ in TgQ226 mice infected with NA CWD (M-CA1) and the associated 2.1 M GdnHCl_1/2_ were intermediate between and distinct from those of NO reindeer and NO moose CWD (*P* ≤ 0.0001 in both cases) (Fig 8A). Analyses of brain homogenates from diseased GtQ226 mice infected with NA or NO CWD prions confirmed the rank stability of PrP^Sc^ conformations recorded in TgQ226 ranging in order from NO reindeer (GdnHCl_1/2_ of R-NO1 = 1.5 M), to NA moose (GdnHCl_1/2_ of M-US1 = 2.0 M), to NO moose (GdnHCl_1/2_ of M-NO1 = 3.5 M) (*P* ≤ 0.0001 in all cases) (Fig 8B). We conclude that CWD strains causing disease in NA cervids, NO reindeer and NO moose are composed of distinct PrP^Sc^ conformers.

To assess whether evolution of M-NO2 to its adapted M-NO2Ad derivative coincided with a change in conformational properties we monitored the denaturation properties of PrP^Sc^ produced in response to infection of Q226 and E226 mice at various passages of infection. For comparison, we first assessed the conformational properties of NA moose CWD prions propagated in Q226 and E226 backgrounds. In accordance with our previous findings showing consistently greater conformational stability of NA deer and elk CWD in Q226 compared to E226 mice [1,38,39], the conformation of NA M-CA1 CWD prions passaged in Q226 (GdnHCl_1/2_ = 2.1 M) was more stable than the same prions passaged in E226 mice (GdnHCl_1/2_ = 1.4 M) (*P* ≤ 0.0001) (Fig 8C). Following passage 2 of M-NO2, denaturation of PrP^Sc^ produced in GtE226 mice with rapid disease onsets generated a comparatively sharp transition between 1 and 2.5 M GdnHCl and a relatively low GdnHCl_1/2_ of 1.8 M (Fig 8D). By contrast, denaturation of PrP^Sc^ produced in GtQ226 mice at the same passage remained incomplete out to 4.5 M GdnHCl, producing a GdnHCl_1/2_ of 2.5 M (*P* ≤ 0.001) (Fig 8D). By passage 3, M-NO2Ad in GtQ226 acquired conformational properties that were distinct from M-NO2 at passage 1 and passage 2, and the denaturation profiles of M-NO2Ad PrP^Sc^ produced in GtQ226 and GtE226 mice resembled those of NA CWD PrP^Sc^ with Q226-PrP^Sc^ also having a more stable response to denaturation than E226-PrP^Sc^ (Fig 8E). Our findings indicate that the conformational properties of PrP^Sc^ change as M-NO2 evolves to form M-NO2Ad during adaptive propagation in CWD susceptible mice.

### Differential infectivity of North American and Norwegian CWD prions and adapted Norwegian moose prions in cultured cells

**Fig 9:**
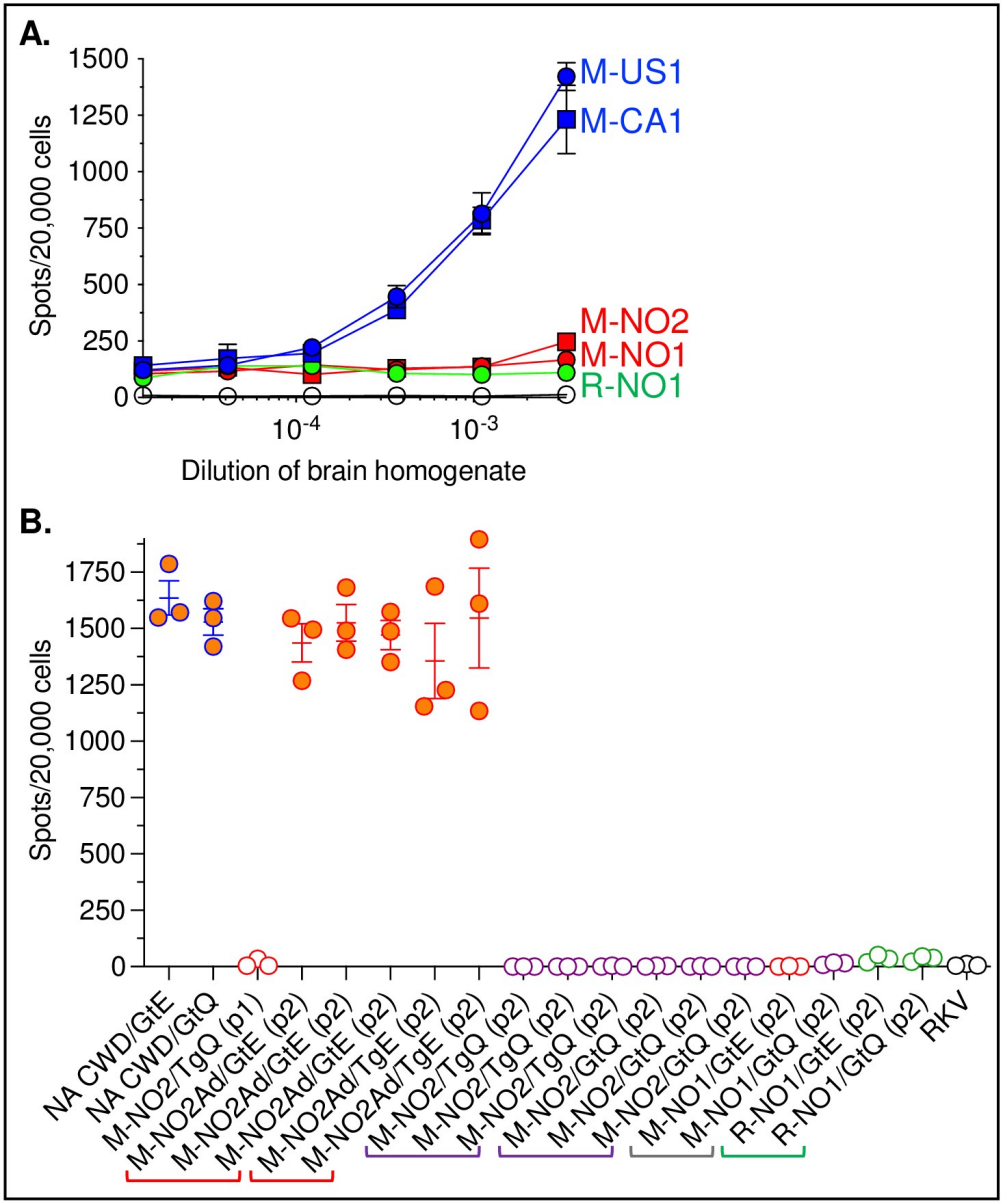
RK-D and RK-E cells are susceptible to North American CWD and adapted NO moose CWD but refractory to Norwegian CWD prions. **A**. RK-D cells were challenged with half-log dilutions of 1% brain homogenates from diseased TgQ226 mice. After four weeks of infection 20,000 cells were filtered onto ELISpot plates, treated with PK, and probed with mAb PRC5 after cells were denatured with guanidine thiocyanate. Error bars, ± SEM of cell counts of infection with brain homogenates from three different infected animals per group. Blue symbols, cell infection with brain homogenates from mice infected with North American moose CWD; red symbols, infection with brain homogenates from mice infected with Norwegian moose CWD; green circles, infection with brain homogenates from mice infected with Norwegian reindeer CWD; grey circles, cells challenged with brain homogenate from *Prnp*^*0/0*^ mice. **B**. RK-E cells were infected in triplicate with individual infected brain homogenates as indicated on the X-axis. ELISpot analyses of each infection were performed after four weeks as described above. RKV, RK13 cells not ectopically expressing PrP resulting from transfection with empty (no PrP coding sequence) expression vector.

In previous studies we showed that rabbit kidney epithelial RK13 cells engineered to express CerPrP^C^ supported the replication of CWD prions from the CNS of diseased NA cervids, and that using cloned counterparts of these CWD-susceptible cells in an approach referred to as the cervid prion cell assay (CPCA) it was possible to study aspects of CWD transmission and to quantify prion titers with sensitivities comparable to bioassay in TgQ226 mice [11,51,52]. Here we found that cloned RK-D cells expressing Q226 previously shown to be susceptible to NA deer and elk CWD [11,51] also responded to dose-dependent infection with NA CWD moose prion isolates M-US1 and M-CA1 which enabled us to estimate titers of 10^6.9^ CPCA units/g of brain tissue for both isolates (Fig 9A). By contrast, RK-D cells were refractory to infection with CWD prions from both NO moose and NO reindeer over the same range of brain homogenate dilutions (Fig 9A). While titer differences between NO and NA CWD cannot be ruled out as a cause of this difference, this explanation seems unlikely in the case of reindeer CWD prions which produced levels of CNS PrP^Sc^ comparable to NA CWD (Fig 6B), and yet failed to produce infection of RK-D over a range of at least 1.5 logs of dilution (Fig 9A). Alternately, the different abilities of NA and NO CWD prions to infect RK-D cells in culture might be a further reflection of strain differences between these prions.

We next compared the abilities of M-NO2 and M-NO2Ad to infect cultured cells. Since M-NO2Ad was detected as a result of passage of M-NO2 in mice expressing E226, we challenged a highly susceptible clone of RK13 cells expressing E226 (RK-E). Prions from brain extracts of multiple diseased GtE226 and TgE226 mice which exhibited rapid kinetics of disease onset following p2 of M-NO2 (Fig 1E), aggregated CNS PrP^Sc^ plaque morphology (Fig 5G and 5H), PrP^Sc^ with altered electrophoretic properties and epitope availability (Fig 6A), and accumulated PrP^Sc^ in the spleens of GtE226 mice (Fig 7C) infected RK-E cells with efficiencies equivalent to those produced by NA CWD prions passaged in either GtE226 or GtQ226 backgrounds (Fig 9B). By contrast, non-adapted M-NO2 prions from TgQ226 mice at passage 1 (Fig 1B) as well as prions from the brains of multiple diseased GtQ226 or TgQ226 mice at passage 2 of M-NO2 (Fig 1E) failed to infect RK-E cells (Fig 9B). RK-E cells were also refractory to CWD prions from brains of GtQ226 and GtE226 at passage 2 of M-NO1 or R-NO1 (Fig 9B). Our findings indicate that whereas RK-D and RK-E cells are refractory to CWD strains causing disease in NO moose and reindeer, conformational adaptation of M-NO2 prions resulted in coincidental acquisition of cell infectability by its adapted descendant, M-NO2Ad.

## Discussion

Our previous analyses of the strain properties of NA deer and elk CWD isolates, most particularly using GtE226 and GtQ226 mice, showed that natural variation at PrP residue 226, which differs among deer, elk and other CWD-susceptible cervids, influences the selection and propagation of prion strains with distinctive disease kinetics, neuropathology, and conformational properties [39]. By extension, these findings raised the prospect that naturally infected NA deer and elk propagate different portfolios of CWD strains [39]. The emergence of CWD in Europe [21–24] raised questions about its etiology and relationship to established NA CWD. The findings reported here showing that prions causing emergent forms of CWD in NO moose and reindeer respond differently to variation at amino acid residue 226 compared to established NA CWD strains provide additional evidence for the influence of residue 226 on CWD strain selection and propagation, and by extension demonstrate that NO moose, NO reindeer and NA cervids are infected with distinct strains. The role of other cervid PrP polymorphisms on strain selection has been addressed using a variety of transgenic mouse models [36,53]. For example, dimorphism at deer PrP residue 95 resulted in the manifestation of two NA CWD strains dictated by the CerPrP primary structure of the recipient host [54]. Studies substantiating the influence of CerPrP primary structural differences are consistent with the effects of additional polymorphisms in human, deer, elk, and sheep PrP on susceptibility to different human and animal prion strains [36,53,55,56]. Collectively, these findings point to a complex relationship between the existence of multiple PrP polymorphic variants in various species and the diversity and evolution of naturally occurring strains. This complex phenomenon is reconciled by the Conformational Selection Model which postulates that prion strains are composed of ensembles of PrP^Sc^ quasi-species conformations, that individual PrP^C^ primary structures have restricted potentials to propagate subsets of maximally fit prion conformations, and thus that strain characteristics reflect the properties of dominant conformer(s) selected for maximized fitness in particular settings [39,51,57–59]. The Conformational Selection Model also clarifies why mice engineered to express PrP primary structures from different hosts faithfully recapitulate the strain properties of prions from those species, while transmission of the same strains to species with different PrP primary structures generally requires adaptation [18,27–29].

The findings reported here also extend our previous collaborative studies that described transmission of NO moose CWD and relatively inefficient primary transmission of NO reindeer prions to BV [44] (Table 2). While results of those studies were consistent with distinctive strain differences between NA and NO CWD, the requirement for adaptation during their experimental passage to this new species presented obvious disadvantages for strain characterization. This drawback is particularly relevant in the case of NO reindeer CWD prions since primary transmission of NO reindeer CWD isolates R-NO1 and R-NO2 in BV resulted in only a single R-NO1-inoculated BV developing disease after ~ 780 days and complete resistance of BV to R-NO2 [44]. Although appropriately cited as evidence for a strain that was distinct from NA CWD [44], the almost uniform resistance of BV upon primary transmission of NO reindeer CWD has to date hindered further characterization of these prions in this model. By contrast, we show that the absence of a transmission barrier to NO reindeer CWD prions in TgQ226, TgE226, GtQ226, and GtE226 mice facilitated characterization of the distinctive properties of this novel emergent prion strain as well as enabling assessment of the transmission properties of lymphoid tissue-derived reindeer CWD prions. More generally, the abrogation of CWD species barriers in Tg and Gt mice expressing CerPrP reproduced the transmission and molecular features of NA and NO CWD strains as they occur in their natural host species, including western blotting patterns and IHC properties of PrP^Sc^. Of particular note, authentic physiological PrP expression in Gt mice enabled the lymphotropic properties of naturally occurring NA and NO CWD strains and adapted derivatives to be recapitulated in this experimental model. Our approach also enabled us to define conformational differences between PrP^Sc^ comprising NO moose, NO reindeer, and NA CWD prion strains as well as differences in the abilities of these strains to infect CWD-susceptible cells in culture.

Our findings also provide important information about CWD strain adaptation. Specifically, the NO moose CWD strain M-NO2, while initially manifesting properties consistent with other emergent NO moose CWD isolates that were different from established NA CWD, acquired multiple characteristics of NA CWD during its adaptive propagation in Tg mice. Acquired characteristics included altered transmission and PMCA responses to the effects of amino acid variation at residue 226; changes in CNS deposition patterns, electrophoretic migration and epitope availability of PrP^Sc^; altered lymphotropism in Gt mice; and acquisition of the ability to infect cell cultures in vitro. Our findings are reminiscent of previous analyses of BSE prions in Tg mice expressing human PrP with M at residue 129 that resulted in a similar bifurcation to produce either a human sporadic CJD or vCJD strain profile [55]. They also fit into a general framework supported by previous studies which suggests that CWD prion conformers are dynamic and evolving, and that strain manifestation is controlled by a critical interplay between CWD prion plasticity and PrP^C^ polymorphisms [60]. Our experiences with M-NO2 and M-NO2Ad are consistent with the notion that CWD, particularly emergent forms of this disease, is in a state of unstable flux in cervid populations. Unlike viruses where natural selection of increasingly fit strains is mediated by nucleic acid mutation, Darwinian evolution of prions is mediated by conformational selection of proteinaceous quasi species [39,51,57–59]. Since adaptation of M-NO2 to M-NO2Ad occurred concurrently with a shift in the conformational status of PrP^Sc^, our findings indicate that M-NO2Ad arose as a result of selection of a PrP^Sc^ conformer that was a minor component of the original quasi-species ensemble constituting M-NO2 which was optimized for propagation in mice expressing E226. Further iterative passages in Q226 and E226 mice revealed that the properties of this selected conformer were consistent with those of NA CWD. In support of the notion that M-NO2 contains strain mixtures, western blotting analyses revealed the presence multiple PK resistant forms of PrP^Sc^ (S1 Fig). Although we discovered that the properties of M-NO2 had adapted as a result of a change in their ability to infect E226 mice, we are currently exploring how the properties of M-NO2 and other isolates are impacted by their consistent passage in Q226 mice. We are also conducting iterative passaging experiments of additional Scandinavian CWD isolates to investigate whether conformational adaptation is a general feature of emergent CWD prion strains.

Our findings provide important information pertaining to risk management of emergent European CWD. First, our in vivo and in vitro findings indicating that strains currently circulating in NO moose preferentially propagate in the presence of Q226 and are relatively restricted by E226 have implications for their potential to preferentially infect cervid species expressing Q226 compared to E226. By contrast, our studies showing that NO reindeer CWD prions propagate with equivalent efficiencies in Q226 and E226 mice suggest the natural host range of NO reindeer CWD may be more expansive than that of NO moose CWD. Currently a single NO red deer homozygous for the E226 allele has been diagnosed with CWD [24] and it will be of considerable interest to ascertain the strain properties of these prions upon transmission to our mouse models. Second, our findings that the lymphotropic profiles of NO moose and reindeer are recapitulated upon transmission to GtQ226 mice and are therefore the result of heritable strain differences between these prions have considerable additional bearing on their relative potentials for natural transmission. Differential PrP^Sc^ accumulation in peripheral tissues, particularly the LRS, appears to be linked to contagious transmission of NA CWD [37]. With this in mind, our findings suggest that diseased NO moose currently propagating non-lymphotropic CWD strains pose a reduced threat for contagious transmission. By contrast, while the strain properties of NO reindeer differ from NA CWD, their shared lymphotropic properties suggest that, like NA CWD, infected NO reindeer pose an increased risk for contagious transmission. Finally, our findings demonstrating adaptation of NO moose M-NO2 prions are relevant when considering the potential for evolution of emergent European CWD. The ability to monitor adaptation of an unstable, incompletely-adapted, emergent NO CWD prion strain into a conformer with properties consistent with those of established NA CWD in an experimental system that authentically models the cardinal features of naturally-occurring CWD underscores the potential for similar conformational adaptation events in natural settings. In light of the multiple shared properties of M-NO2Ad with established NA, we speculate that this newly adapted strain corresponds to established NA CWD and therefore likely shares the feature of contagious transmission. However, while our determination of similarity makes use of multiple criteria, the possibility that M-NO2Ad has additional as yet undetected characteristics that distinguish it from NA CWD, for example improved capacities to infect other animal species and humans, cannot be excluded. Nonetheless, our findings raise the possibility that the continued propagation of currently unstable, emergent European CWD strains, particularly in cervid hosts with different codon 226 PrP genotypes, will ultimately result in the generation of adapted derivatives with properties resembling those of established NA CWD by a process of natural conformational selection. Regardless of the etiology of NO CWD and other emergent cases in Sweden and Finland, the unlikely possibility that stochastic emergence of unstable, incompletely adapted strains will remain confined to Scandinavia argues for prudential CWD surveillance in additional European countries.

By extension, our findings describing adaptation of novel, unstable, emergent NO CWD strains raise questions about the etiology of NA CWD. Two mutually exclusive hypotheses have been proposed to account for the origins of NA CWD. The first suggests that established NA CWD strains resulted from spontaneous prion formation following sporadic conversion of cervid PrP^C^ to PrP^Sc^. The spontaneous generation hypothesis derives support from structural studies that identified a well-defined (rigid) loop between the β2 strand and α-helix 2 of cervid PrP, with suggestions that this structural feature contributed to the contagious properties of CWD [61]. In accordance with this proposal, Tg mice overexpressing a mouse PrP construct with the two amino acids that define loop rigidity, and Tg mice expressing BV PrP, which also contains a rigid loop [62], spontaneously developed transmissible diseases [63,64]. We showed in previous studies that E and Q variations at residue 226 affected hydrogen bonding interactions that modulate plasticity of a solvent-accessible discontinuous epitope formed between the distal region of α-helix 3 and residues in the β2–α2 loop [36]. It seems likely therefore that this structural feature of residue 226 contributes to a process that modulates strain-dependent conversion efficiency of PrP^C^ to PrP^Sc^. An alternate hypothesis posits that CWD resulted from exposure to prions from sympatric species that subsequently adapted for optimal propagation in cervids. Support for this notion derives from demonstrated examples of interspecies prion transmission and coincident adaptation. In particular, recent studies in bovine PrP expressing Tg mice indicated that challenge with Nor98/atypical scrapie prions resulted in adaptation to the classical BSE agent [65]. Our findings describing selective adaptation of M-NO2 to M-NO2Ad support the notion that NA CWD may have similarly originated from an unstable progenitor(s) that evolved by conformational adaptation to produce increasingly fit strains in cervids of different codon 226 PrP genotypes. It seems likely that continued experimental studies in Gt mice will be a valuable means to address the origins of CWD.

Our findings in which we compared CWD transmissions in Tg and Gt mice also provided insights into the importance of precisely controlled physiological PrP expression for facilitating prion transmission and revealed unexpected capacities of Gt mice for studying the properties of prion strains including their lymphotropic properties and potentials for selective adaptation. While early studies in Tg mice supported a simplified view which described an inverse relationship between levels of transgene-encoded PrP expression in the CNS and the kinetics of disease onset and [46,47], subsequent findings revealed a more complex picture which supported the importance of prion replication in lymphoid tissue for certain strains [66]. Additional studies in Tg mouse models expressing different levels of PrP indicated that PrP gene dosage also influences strain evolution [67]. The findings reported here in concert with those previous reports suggest that standard ic challenges of Tg mice, particularly with lymphotropic strains, may artificially skew the portfolio of available conformers and ultimately provide misleading estimates of the potential for interspecies prion transmission. The ability to recapitulate the properties of lymphotropic CWD strains in Gt mice also provides insights into the unexpectedly less efficient transmission of certain CWD strains in overexpressing Tg mice. It seems likely that the improved susceptibility of Gt mice to NO reindeer CWD prions is linked to the lymphotropic properties of this strain. These findings build on our previous observation that Tg mice overexpressing deer PrP in the CNS are relatively resistant to peripheral inoculations with NA CWD prions, most likely as a result of sub-optimal splenic prion replication [39]. We note, however, that the transmission studies of NO CWD reported here were performed by ic challenges of mice. While this process most likely also results in at least some dissemination of prion infectivity to peripheral compartments through blood, the outcomes of ongoing experiments involving challenges Gt mice with lymphotropic and non-lymphotropic NO and NA CWD strains by a variety of infection routes, including ip challenges, will be of considerable interest.

Finally, the emergence of novel Scandinavian CWD strains, their relative instability, and now proven potential for adaptation increase uncertainties about CWD zoonosis. The highly efficient peripheralization of CWD in NA deer and elk and resultant prion infectivity in skeletal muscle and other materials destined for consumption by humans [9,35], their ability to template in vitro conversion of human PrP [68,69], and the emerging results of transmission studies in non-human primates [70–72] elicit additional concern. As alluded to above, our findings in GtE226 and GtQ226 mice detailing the importance of peripheral PrP expression force us to reconsider the precision of Tg mice expressing human PrP for assessing zoonotic transmission of CWD [73–76] and suggest that Gt models provide an improved platform for making this determination. Unfortunately, previously generated knock-in mice designed to be susceptible to human prions had long incubation times and incomplete attack rates; similarly constructed knock-in mice designed to be susceptible to BSE were paradoxically less responsive to this strain than their wild type mouse counterparts [77,78]. These findings led to suppositions that the poor susceptibly of knock-in models reflected inadequate PrP expression compared to Tg counterparts. The responses of GtE226 and GtQ226 mice to CWD, which are in some case superior to overexpressing Tg counterpart mice, reveal that is not the case. These discrepancies therefore raise questions about the underlying causes for the differences in susceptibility of GtQ226 and GtE226 mice compared to previous knock-in models. One possible explanation rests on PrP primary structural considerations. While GtE226 and GtQ226 mice express PrP coding sequences that include cervid N-terminal signal peptide (SP) sequences [39], their poorly responsive predecessors expressed coding sequences engineered to contain the mouse PrP N-terminal SP [77–79]. Amino acid substitutions in the N-terminal SP have considerable impacts on prion pathogenesis in other settings [79–82], and certain CWD susceptibility polymorphisms in deer PrP map to this location [83]. Primary structural differences between mouse and cervid SP coding sequences therefore offer a parsimonious explanation to account for the poor responses of previously engineered models. We therefore anticipate that Gt mice expressing cognate human PrP coding sequences including the human PrP N-terminal SP will have accurate susceptibilities to human prions on a par with the responses of GtE226 and GtQ226 mice to CWD, and that they will provide a refined and improved means to assess the zoonotic potential of CWD strains and their adapted derivatives.

## Materials and methods

### Ethics statement

All animal work was performed in an Association for Assessment and Accreditation of Laboratory Animal Care International accredited facility in accordance with the Guide for the Care and Use of Laboratory Animals. All procedures used in this study were performed in compliance with and were approved by the Colorado State University Institutional Animal Care and Use Committee.

### CWD inocula

The moose isolate referred to as M-US1 represents the seminal report of CWD in NA moose resulting from experimental oral inoculation of captive moose with a pooled preparation of deer CWD prions [33]. Moose isolates M-US2 and M-US3 [84] were naturally occurring, subclinical cases killed by hunters in northcentral Colorado. The moose isolate referred to as M-CA1 was the first naturally occurring case of CWD in a Canadian moose detected in 2013 [23]. NO moose and reindeer CWD isolates have been previously described [21–23,44].

### Animal models

The development and characterization of TgQ226, TgE226, GtQ226, and GtE226 mice has been described previously [29,35,39]. For inoculation of mice, 10% homogenates of brain or lymphoid tissues from CWD affected moose, reindeer, deer, or elk were prepared by mechanical disruption (MP Biomedical, Irvine, CA) in phosphate-buffered saline (PBS) lacking calcium and magnesium ions. Equal numbers of male and female mice between the ages of four to six weeks were anaesthetized with halothane and ic inoculated freehand with 30 μl of 1% brain or lymphoid tissue homogenates into the right parietal lobe using a 26-gauge needle at a depth of ~ 2 mm. All animals were subsequently monitored three times a week for the development of neurological signs indicative of prion diseases. These signs included truncal ataxia, loss of extensor reflex, slowed movement, wobbling/flattened gait, plastic tail, dorsal kyphosis, head bobbing, and rough coat. The time to disease onset, or incubation period, is defined as the time between inoculation and the first day on which subsequently progressive clinical signs were identified. Unless stated otherwise, all animals for which a clinical diagnosis was made were confirmed to have died as a result of prion infection by analysis of PrP^Sc^ in CNS material by various means.

### Histoblot analysis

Histoblots were prepared and analyzed as previously described [48]. In brief, whole brains were snap frozen on dry ice. Ten μm coronal cryostat sections on slides were transferred to nitrocellulose membranes which were treated with 0.2 mg/ml proteinase K (PK) for 1 h at 37°C. Membranes were incubated with a 2 mM solution of phenylmethylsulfonyl fluoride (PMSF) for 15 minutes. Membranes were placed in 3M guanidine isothiocyanate for 10 minutes at room temperature and then blocked in 5% blotto for 30 minutes at room temperature followed by overnight incubation at 4°C with mAb PRC5 at a dilution of 1:2,500. Alkaline phosphatase conjugated goat anti mouse IgG (Soutern Biotech, 1030–04) was then applied for 1 h at room temperature at a dilution of 1:2,500. Membranes were developed using 5-bromo-4-chloro-3-indolyl phosphate (BCIP)/nitro blue tetrazolium (NBT) (Sigma Aldrich, 11697471001) for 5–15 minutes. Images were captured using a Nikon Z1000 microscope.

### Immunohistochemical analyses

IHC was performed as previously described [85]. In brief, slides were heated to 60°C for 30 minutes prior to xylene and graduated ethanol treatment followed by treatment with 88% formic acid for 30 minutes. Antigen retrieval was then performed in the 2100 Retriever (ProteoGenix, Schiltigheim, France) using citrate buffer followed by endoperoxidase quenching in 3% hydrogen peroxide. Slides were blocked in 5% blotto for 30 minutes at room temperature before overnight incubation at 4°C with primary antibody D18 at a 1:2,500 dilution. Slides were exposed to biotin labelled goat Fab anti-human IgG secondary antibody (Southern biotech, Birmingham, AL) at a 1:2,500 dilution for 1 h at room temperature, then developed for 30 minutes at room temperature utilizing avidin-conjugated horseradish peroxidase (HRP) with diaminiobenzidine (DAB) as substrate (Vector Laboratories, Burlingame, CA). Slides were counterstained with hematoxylin, run through graduated ethanol treatment, cover slipped, and imaged at 40x or at 100x under oil immersion.

### Analysis of PrP^Sc^ by western blotting

Protein concentrations in 10% brain homogenates were determined by bicinchoninic acid assay (BCA) (Piece Biotechnology, Rockford, lL, USA). Homogenates were treated with 50 μg/ml PK (Roche, Mannheim, Germany) in the presence of 2% sarkosyl for 1 h at 37°C. Digestion was terminated with PMSF at a final concentration of 2 mM. Prior to electrophoresis samples were boiled in XT-sample buffer (Bio-Rad Laboratories, Hercules, CA) in the absence of reducing agents for 5 min and were loaded onto precast 12% discontinuous Bis-Tris gels (Bio-Rad Laboratories, Hercules, CA). Electrophoretically separated proteins were transferred overnight to PVDF-FL membranes (Millipore, Billerica, MA, USA). Membranes were blocked for 1 hour in 5% non-fat milk in TBS-T, and then probed with PRC5 and PRC1 mAbs, followed by HRP–conjugated anti-mouse IgG secondary antibody. Membranes were developed using ECL 2 Western blot substrate (Thermo Scientific, USA). Ten percent homogenates (w/v) of frozen spleen tissues in PBS lacking calcium and magnesium ions and containing protease and phosphatase inhibitors (Sigma Aldrich, St. Louis, MO) were produced by mechanical disruption (MP Biomedical, Irvine, CA) using zirconium oxide beads (Next Advance, Troy, NY). Total protein concentration was determined by BCA assay (Pierce Biotechnology, Rockford, IL). Spleen homogenates (1 mg) were treated with 1 mg/ml DNase I (Sigma Aldrich, St. Louis, MO) and 5 mM MgCl2 for 1 h at 37°C with gentle agitation. DNase-treated spleen homogenates were adjusted to 2% sarkosyl and treated with 50 μg/ml PK for 1 h at 37°C with gentle agitation. PK digestion was terminated with 2 mM PMSF for 10 min at room temperature. Samples were subjected to ultracentrifugation at 100,000g for 1 hour at 4°C in a Beckman Coulter Optima Max-XP ultracentrifuge (Beckman Coulter, Indianapolis, IN). Pellets were suspended in 50 μl SDS-PAGE sample loading buffer, heated at 100°C for 10 minutes, and subjected to SDS-PAGE and western blotting with chemiluminescent detection.

### Protein misfolding cyclic amplification

PMCA was performed as previously described [86]. Homogenized CWD-infected brain materials were used to initiate the PMCA seeding reaction. This mixture was considered to be the 10^−2^ sample in the dilution series, and was serially diluted in 10-fold increments to 10^−14^ in uninfected brain homogenates from TgQ226 and TgE226 mice. These 10% brain homogenates were prepared by manual homogenization in conversion buffer (1X phosphate-buffer saline, 150 mM sodium chloride, 1% Triton X-100) with the addition of protease inhibitors. Prior to PMCA, 10% brain homogenates were supplemented with 12 mM EDTA and 0.05% digitonin. These reaction conditions were held constant across each PMCA experiment. PMCA was performed using a QSonica700 sonicator at amplitude 20-35A and subjected to one round of PMCA (144 sonication/incubation cycles).

### Conformational stability assay

Conformational stability assay was conducted as previously described [11,39,51,56]. Briefly, brain homogenates containing 5 μg to 30 μg protein were incubated with various concentrations of GdnHCl in 96-well plates for 1 h at room temperature. Samples were adjusted with PBS to 0.4 M GdnHCl and transferred onto nitrocellulose (Whatman GmbH, Dassel, Germany) using a dot blot apparatus. After two PBS washes, the membrane was air-dried for 1 hour, then incubated with 5 μg/ml PK in cell lysis buffer (50 mM Tris-HCl, pH 8.0, 150 mM NaCl, 0.5% sodium deoxycholate, 0.5% Igepal CA-630) for 1 hour at 37°C. PK was inactivated with 2 mM PMSF. Membranes were denatured in 3 M guanidine thiocyanate in Tris-HCl, pH 7.8 for 10 minutes at room temperature. After four washes with PBS, the membrane was blocked with 5% nonfat milk in TBST for 1 hour and probed overnight at 4°C with mAb PRC5 (15) at a dilution of 1∶5000, followed by HRP-conjugated goat anti-mouse IgG secondary antibody. The membrane was developed with ECL Plus and scanned with an ImageQuant LAS 4000 (GE Healthcare), and signals analyzed with ImageQuant TL 7.0 software (GE Healthcare).

### Cell-based prion infections

RK-D and RK-E cells [11] were cultured in DMEM containing 10% (v/v) calf serum and 1 μg/ml penicillin/100 U/ml streptomycin. Cells were passaged at a ratio of 1:10 every five days. Cells were infected as described previously [52] with the following modifications adapted from [87]. Wells of 96-well plates were coated with various dilutions of brain homogenates infected with either NA or NO CWD prions in a final volume of 100 μl/PBS for 1 h. Remaining liquid was removed by aspiration and wells were washed twice with 150 μl PBS and air dried. Twenty thousand cells were added to each pre-coated well in a volume of 100 μl culture medium. Cells were maintained in this state for four weeks, and 150 μl cell culture medium was changed very five days. After this time cells were removed by treatment with trypsin, and 20,000 cells were filtered onto ELISpot plates and analyzed as described previously [52]. RK13 cells stably transfected with pIRESpuro3 vector lacking a PrP coding sequence, referred to as RKV, produce no PrP^C^ and served as negative controls for prion infections.

### Statistical information

Statistical analyses were performed using Graphpad Prism software (San Diego, CA). Statistical significance between survival curves of inoculated groups was assessed by comparing median times of survival of various inoculated groups using the log rank (Mantel-Cox) test. Statistical significance between denaturation curves of PrP^Sc^ was assessed by comparing log EC_50_ values.

## Supporting information

S1 Fig Additional comparisons of PrP^Sc^ in the CNS of animals infected with moose CWD prions from North America and Norway. Western blot analysis of PrP^Sc^ in the CNS of diseased NO and NA moose (**A**.) and TgQ226 mice (**B**.), referred to in figure captions as TgQ. **C**., PrP^Sc^ quantification in TgQ226 and GtQ226 mice. Columns 1, 2: uninfected TgQ226; 3, TgQ226 infected with M-US1; 4, TgQ226 infected with M-CA1; 5, TgQ226 infected with M-NO1; 6, TgQ226 infected with M-NO2; 7, uninfected GtQ226; 8, GtQ226 infected with M-US1; 9, GtQ226 infected with M-NO1; 10, GtQ226 infected with M-NO2. Error bars, ± SEM of samples from three animals, each analyzed in triplicate. **D**., immunoblot of PrP^Sc^ from Norwegian moose CWD prions passaged in TgQ226. Red arrows to the side of blots in **A**., and **D**. indicate the position of the additional lower molecular weight non-glycosylated PrP^Sc^ fragment associated with infection with M-NO2 and M-NO3 CWD prions. The position of molecular weight markers approximating 36- and 29-kDa are shown to the left of blots.
(TIF)
