# Supplementary figures and images for: Adaptive selection of a prion strain conformer corresponding to established North American CWD during propagation of novel emergent Norwegian strains in mice expressing elk or deer prion protein
Source: PLoS Pathog. 2021 Jul 26;17(7):e1009748. doi: 10.1371/journal.ppat.1009748 (PMC8341702; doi:10.1371/journal.ppat.1009748)

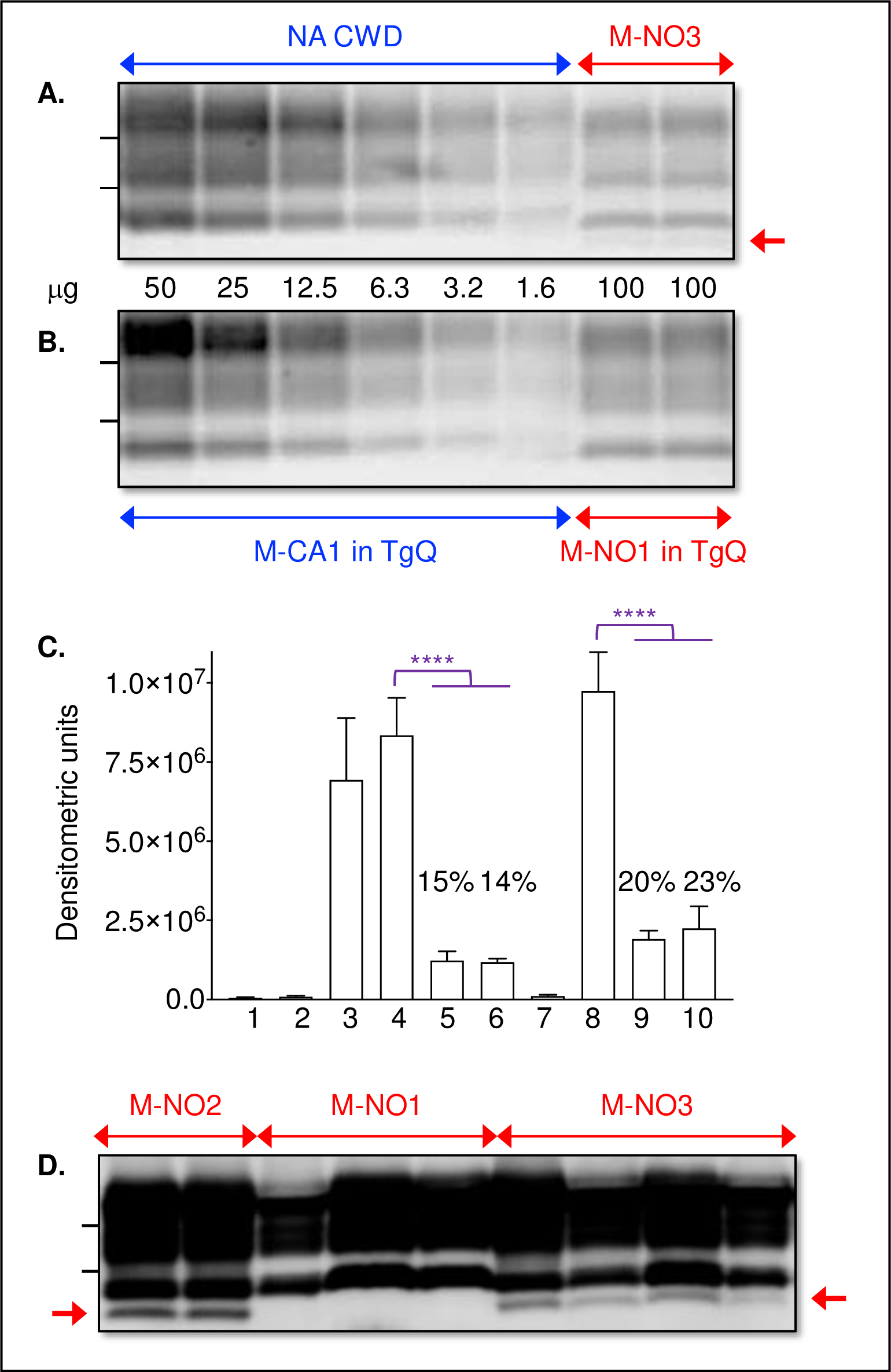

Supplement: S1 Fig — Western blot analysis of PrPSc in the CNS of diseased NO and NA moose (A.) and TgQ226 mice (B.), referred to in figure captions as TgQ. C., PrPSc quantification in TgQ226 and GtQ226 mice. Columns 1, 2: uninfected TgQ226; 3, TgQ226 infected with M-US1; 4, TgQ226 infected with M-CA1; 5, TgQ226 infected with M-NO1; 6, TgQ226 infected with M-NO2; 7, uninfected GtQ226; 8, GtQ226 infected with M-US1; 9, GtQ226 infected with M-NO1; 10, GtQ226 infected with M-NO2. Error bars, ± SEM of samples from three animals, each analyzed in triplicate. D., immunoblot of PrPSc from Norwegian moose CWD prions passaged in TgQ226. Red arrows to the side of blots in A., and D. indicate the position of the additional lower molecular weight non-glycosylated PrPSc fragment associated with infection with M-NO2 and M-NO3 CWD prions. The position of molecular weight markers approximating 36- and 29-kDa are shown to the left of blots. (TIF) [file ppat.1009748.s001.tif]
